# Experimental Study on the Axial Compressive Behavior of an Aluminum Alloy Foam Concrete Composite Column

**DOI:** 10.3390/s26175361

**Published:** 2026-08-25

**Authors:** Bo Yang, Ao Zhang, Ronghua Su, Jian He, Xinyi Zhang, Zixun Wu

**Affiliations:** 1 College of Aerospace and Civil Engineering, Harbin Engineering University, Harbin 150001, China; yangbo2023@hrbeu.edu.cn (B.Y.); hejian@hrbeu.edu.cn (J.H.); wuzixun0325@foxmail.com (Z.W.); 2 Institute of Defense Engineering, AMS, PLA, Beijing 100850, China; suronghua2023@163.com (R.S.); zxy002003@163.com (X.Z.)

**Keywords:** prefabricated construction, foam concrete, composite column, carrying capacity, axial compressive tests

## Abstract

**Highlights:**

**What are the main findings?**

- Axial compression tests showed that cracks mainly initiated and propagated along the diamond-shaped diagonal-braced surfaces, with the middle column segment being the most vulnerable region.
- Strain analysis indicated that foam concrete dominated the early-stage axial resistance, while the semi-passive confinement effect of the aluminum alloy frame weakened as the load increased.

**What are the implications of the main findings?**

- The diagonal-braced region and middle column segment should be considered critical locations in the design and damage evaluation of aluminum alloy–foam concrete composite columns.
- Finite element results suggest that, within the investigated numerical range, two interlocking keys, zero cover thickness, and a 3 mm frame thickness can improve synergistic load-sharing and axial capacity.

**Abstract:**

A lightweight and high-strength structural system is increasingly demanded in prefabricated and sustainable construction; however, accurately evaluating the mechanical behavior of novel composite members remains challenging. This study proposes an aluminum alloy foam concrete composite column consisting of an embedded aluminum alloy frame and foam concrete infill. Material tests, a full-scale axial compression test, and finite element-based parametric analyses were conducted to verify the hypothesis that composite action between the embedded aluminum alloy frame and foam concrete governs the axial load-transfer mechanism and bearing capacity of the proposed column. The results show that initial cracking mainly occurred in the middle region of the column along the diagonal brace direction, while the composite column maintained favorable elastic performance under relatively high load levels. The aluminum alloy frame provided a semi-passive confinement effect on the foam concrete, although this effect gradually weakened with increasing load due to lateral deformation of the diagonal braces. Moreover, an appropriate arrangement of interlocking keys significantly improved the composite action and overall stability of the column. These findings provide experimental and numerical evidence for assessing the axial compression performance of aluminum alloy foam concrete composite columns and offer guidance for the design and optimization of this novel lightweight structural system.

## 1. Introduction

With the rapid development of prefabricated construction and green building concepts, lightweight, efficient, and sustainable structural systems have attracted increasing attention in the engineering community [1]. Aluminum alloys have been increasingly used in structural engineering because of their low density, corrosion resistance, and favorable fabrication performance. Mazzolani [2] discussed the application potential of three-dimensional aluminum structures, while Georgantzia et al. [3] and You et al. [4] provided comprehensive reviews on the mechanical behavior, design issues, and engineering applications of aluminum alloy structural members. Recent studies have further extended aluminum alloys to composite structural members. Chen et al. [5] investigated the axial compressive behavior of concrete-filled CFRP–aluminum alloy tubular columns, whereas Yan et al. [6] proposed a theoretical model for circular concrete-filled aluminum alloy tubular short columns. In addition to member-level studies, Guo et al. [7] examined the hysteretic behavior of aluminum alloy gusset joints, and Liu et al. [8] summarized the mechanical performance of aluminum alloy grid structures. Lightweight cementitious materials have also been widely investigated for reducing structural self-weight and improving functional performance. Li et al. [9] focused on the fracture behavior of bamboo fiber-reinforced lightweight aggregate concrete, while Wang et al. [10], Zhou and Brooks [11], and Chang et al. [12] mainly examined the thermal insulation and thermomechanical properties of lightweight cementitious composites. Wang et al. [13] further studied the shrinkage and strength development of coal gangue ceramsite lightweight aggregate concrete. At present, this structural form has been implemented in railway interval facilities, training units, and emergency housing, demonstrating high assembly efficiency and strong economic advantages [14].

Recent studies on cold-formed steel–foam concrete composite columns indicated that foam concrete infill can suppress local buckling and improve axial bearing capacity [15]. Other studies focused on the influence of pore structure, fiber addition, and reinforced grid confinement on the mechanical properties of foam concrete or foam concrete-filled members [16,17]. Nevertheless, these studies are mainly related to steel-based composite columns, concrete-filled aluminum alloy tubes, or material-level foam concrete behavior. Limited attention has been paid to aluminum alloy spatial-frame foam concrete composite columns, especially members with X-shaped diagonal braces and interlocking keys. For this type of member, the load-transfer mechanism between the aluminum alloy frame and foam concrete, the cracking characteristics around the diagonal braces, and the influence of key structural parameters remain unclear. Foam concrete is a lightweight porous cement-based material produced by introducing stable air voids into a cementitious slurry. Compared with normal concrete, foam concrete has lower density, better flowability, improved thermal insulation, and favorable energy absorption capacity. These characteristics make it attractive for prefabricated and lightweight structural systems. However, the mechanical properties of foam concrete are strongly dependent on its dry density, pore structure, curing condition, and testing age. In general, reducing the density of foam concrete can effectively decrease structural self-weight, but it may also reduce compressive strength and elastic modulus. Therefore, when foam concrete is used in load-bearing composite members, its interaction with the surrounding structural frame becomes critical. Aluminum alloys are also widely used in lightweight structural systems because of their low density, corrosion resistance, extrusion formability, and high prefabrication efficiency. Compared with steel, aluminum alloys have a lower elastic modulus, which makes local stability and connection design particularly important. In composite structural members, the aluminum alloy frame can provide the main load-bearing skeleton, while its geometric configuration and connection details directly affect stiffness, load transfer, and failure development. Combining foam concrete with an aluminum alloy frame provides a potential way to develop lightweight composite columns with improved structural efficiency. Therefore, the scientific issue addressed in this study is whether an embedded aluminum alloy spatial frame and foam concrete can develop effective composite action under axial compression, and how this interaction is affected by the interlocking keys, concrete cover thickness, and aluminum alloy frame thickness. However, the axial load-transfer mechanism and failure characteristics of aluminum alloy–foam concrete composite columns remain insufficiently clarified, particularly for members in which an embedded aluminum alloy frame is connected to foam concrete through interlocking keys. The primary hypothesis of this study is that the aluminum alloy frame and foam concrete can develop composite action under axial compression, and that this composite action is governed mainly by the interfacial mechanical connection and the relative stiffness contribution of the aluminum alloy frame. To test this hypothesis, one full-scale axial compression test was conducted to identify the failure mode, load–displacement response, and strain development of the proposed composite column. The hypothesis was evaluated using the following criteria: (1) whether the observed cracking pattern corresponds to the stress concentration regions predicted by the finite element model; (2) whether the measured longitudinal and transverse strain responses indicate coordinated load transfer between the aluminum alloy frame and foam concrete; and (3) whether the finite element model can reproduce the experimental load–displacement and strain responses with acceptable accuracy. After validation, the finite element model was used to investigate the effects of concrete cover thickness, number of interlocking keys, and aluminum alloy frame thickness on the axial bearing capacity within the selected parameter range.

## 2. Materials and Methods

### 2.1. Test Design

The aluminum alloy foam concrete composite column investigated in this study has a total height of 2.4 m and is composed of foamed concrete, an aluminum alloy frame, X-shaped diagonal braces, M10 bolts, and 300 mm aluminum strips.

**Figure 1:**
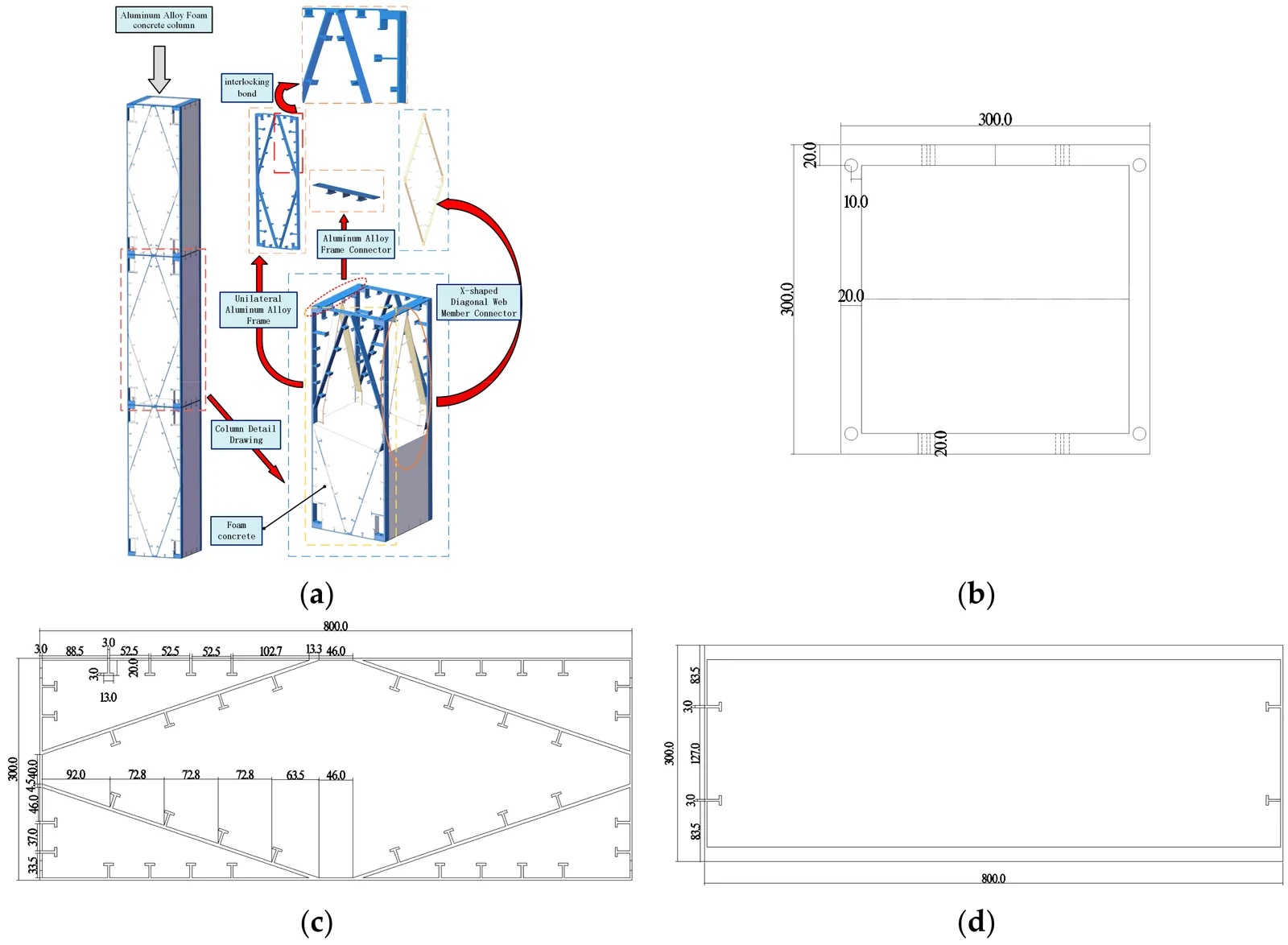
Configuration of the composite column. (**a**) Schematic of the composite column; (**b**) side view; (**c**) front view; (**d**) top views.

Each column is formed by welding two aluminum alloy frames together using 300-mm aluminum strips and X-shaped braces, while the three column segments are connected by bolts. In addition, T-shaped anchorage components are incorporated into the aluminum alloy frame and X-shaped braces to enhance the interfacial bond between the foamed concrete and aluminum alloy. The configuration and detailed dimensions of the components are illustrated in Figure 1.

The dimensions of the column and aluminum alloy frame were determined based on the prototype prefabricated composite column used in the proposed structural system, while also considering laboratory loading capacity, fabrication feasibility, and the requirement for arranging strain gauges and displacement transducers. The column height of 2.4 m corresponds to the typical height of a modular wall-column unit. The 300 mm aluminum strips and M10 bolts were adopted to ensure reliable segmental connection and convenient assembly of the aluminum alloy frame. The X-shaped diagonal braces were selected to improve the lateral stability of the aluminum alloy frame and to provide an additional diagonal load-transfer path under axial compression. This configuration can help restrain local deformation of the vertical aluminum members and enhance the interaction between the frame and the foam concrete. The T-shaped anchorage components were not used as primary braces, but as mechanical interlocking components embedded in the foam concrete. Their purpose was to increase the interfacial bond and shear resistance between the aluminum alloy frame and the foam concrete, thereby reducing relative slip and improving composite action.

### 2.2. Mechanical Properties of Materials

According to Foamed Concrete (JG/T 266-2011) and Technical Specification for Application of Foamed Concrete (JGJ/T 341-2014) [18,19], compressive strength tests were conducted on 100 mm × 100 mm × 100 mm standard cube specimens prepared from the same batch as the full-scale composite column. Three cube specimens were tested to determine the compressive strength of the foam concrete used in this study. The tensile tests of the aluminum alloy specimens were conducted using a universal testing machine (Instron 5982, Norwood, MA, USA) equipped with a 100 kN load cell.

The foam concrete used in this study was prepared using the physical foaming method, because the target density can be controlled by adjusting the foam content. First, the cement-based slurry was prepared by mixing cement, water, and admixtures until a uniform mixture was obtained. Then, preformed foam generated from a foaming agent was gradually added to the slurry and mixed at a low speed to avoid foam collapse. The fresh foam concrete was poured into the aluminum alloy frame without mechanical vibration, because excessive vibration may damage the pore structure. The cube specimens and the full-scale composite column were prepared from the same batch of foam concrete. The target dry density of the foam concrete was 800 kg/m^3^, and the measured dry density was 763 kg/m^3^. After casting, the specimens were covered with plastic film for the first 24 h and then cured until the test age. The cube compressive strength test and the axial compression test of the composite column were conducted at an age of 28 days.

**Figure 2:**
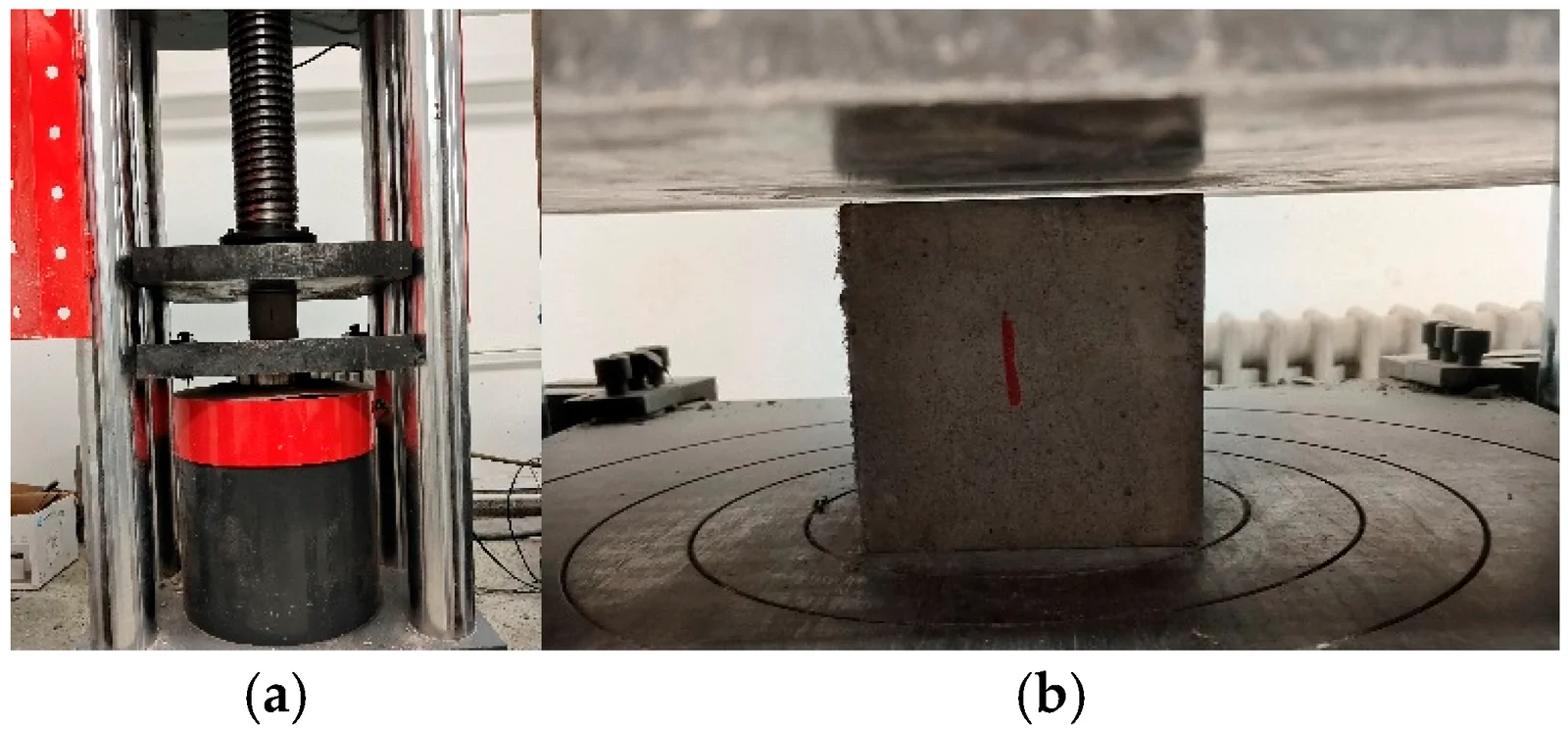
Foam concrete axial compression material test. (**a**) Axial compression test; (**b**) foam concrete cubic specimens.

To characterize the mechanical properties of the composite column, axial compression tests were conducted on cubic specimens with a side length of 100 mm using a uniaxial compression testing machine. The testing configuration is illustrated in Figure 2. In this study, 5 MPa was adopted as the minimum target compressive strength of the foam concrete infill to ensure that the foam concrete could participate in axial load transfer and provide a certain degree of lateral restraint and integrity enhancement to the embedded aluminum alloy frame. Based on the axial compression results obtained from the three specimens, the characteristic axial compressive strength of the foam concrete is determined to be 6.98 MPa.

**Figure 3:**
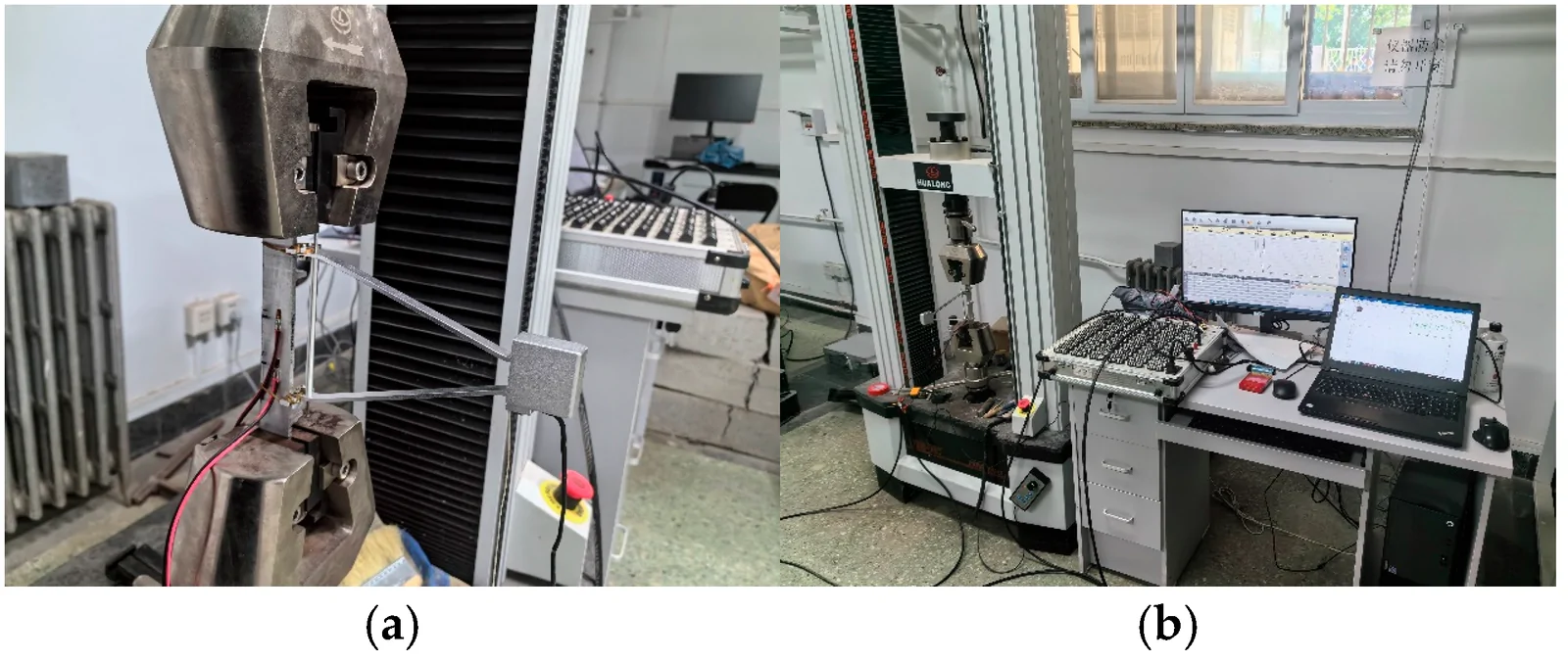
Instrumentation layout and test setup. (**a**) Test setup; (**b**) instrument layout.

According to Metallic materials-Tensile testing [20], the chord members of the extruded aluminum alloy frame were cut into standard tensile specimens with a cross-sectional dimension of 4 mm × 25 mm, and a total of six specimens were prepared. Tensile deformation was measured using both an extensometer and electrical resistance strain gauges. The extensometer was directly connected to the testing machine, allowing the yield strength to be computed automatically by the accompanying software. For the strain-gauge measurements, the strain data collected by a Donghua 3816N acquisition system were synchronized with the load data exported from the testing machine The specimen arrangement is shown in Figure 3.

The aluminum alloy frame used in this study was made of 6005A-T6 aluminum alloy. This alloy was selected because of its good extrusion formability, relatively high strength, and favorable corrosion resistance, making it suitable for prefabricated lightweight structural members. The nominal chemical composition of 6005A-T6 aluminum alloy is listed in Table 1.

The measured results are summarized in Table 2, where the yield strength corresponds to the 0.2% offset yield strength *f*_0.2_. As shown in Table 1, the 0.2% offset yield strength obtained from the extensometer ranged from 210 to 225 MPa, with an average value of 215 MPa. The yield strength obtained from the strain gauges was approximately 230 MPa. The difference between the two measurement methods was about 15 MPa, corresponding to approximately 7.0% of the extensometer-based average value. This discrepancy can be attributed to the different strain measurement mechanisms. The extensometer measures the average axial deformation over a specified gauge length and is directly connected to the testing machine, whereas the strain gauges record local surface strain at selected measurement points. Therefore, the strain-gauge results may be influenced by local strain concentration, bonding quality, slight eccentricity during loading, and synchronization between the load data and the strain acquisition system. Considering the relatively small difference between the two methods, the discrepancy has limited influence on the evaluation of the mechanical behavior of the composite column. For conservative analysis, the extensometer-based average yield strength of 215 MPa was adopted as the representative yield strength of the aluminum alloy in the subsequent analysis and finite element model.

### 2.3. Test Setup and Loading Protocol

#### 2.3.1. Test Setup

**Figure 4:**
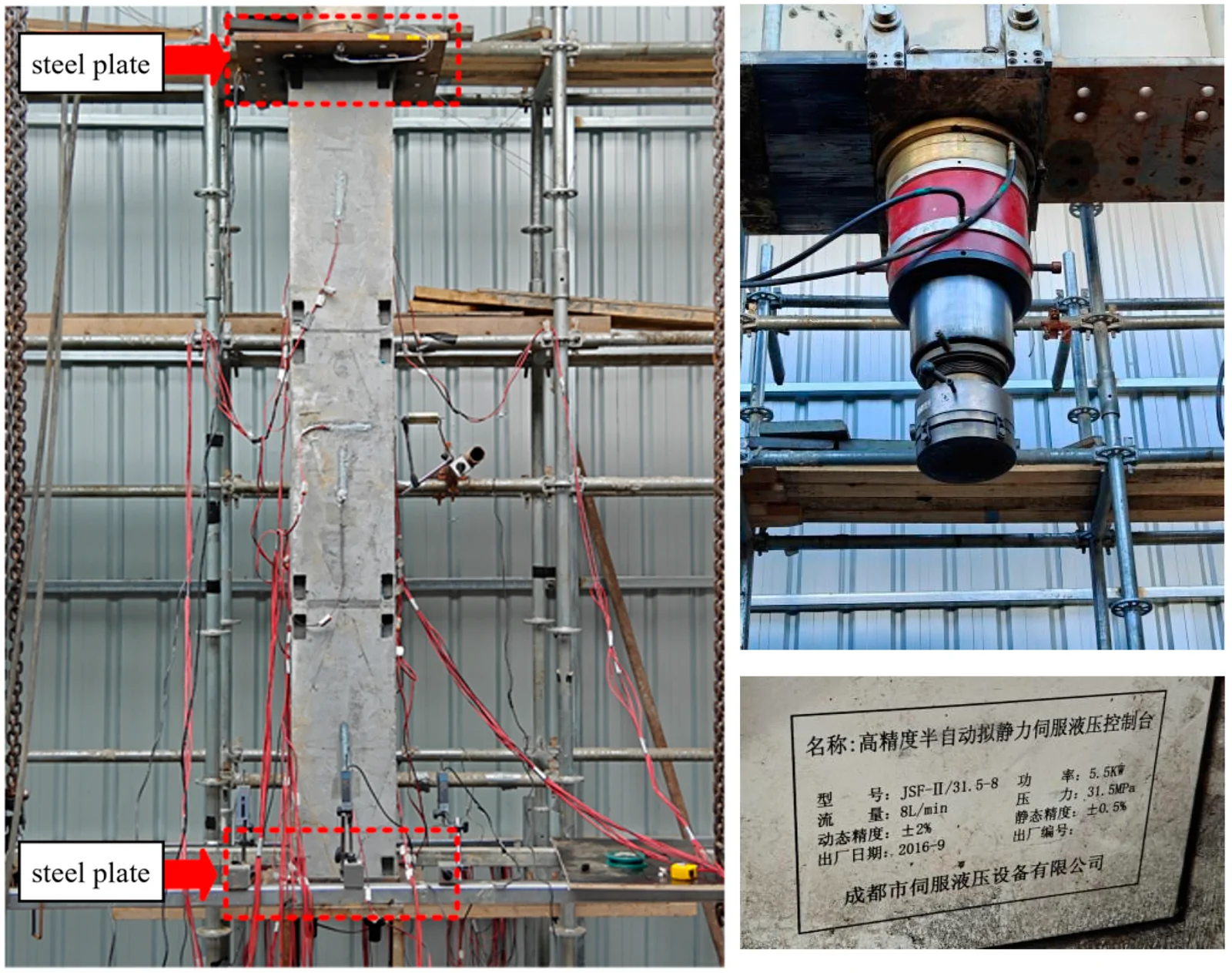
Test Loading Apparatus. Note: Test equipment: high-precision semi-automatic hydraulic servo force closed-loop controller (JSP-11/31.5-8, Chengdu Tongzhen Hydraulic Equipment Co., Ltd., Chengdu, China).

The axial compression loading setup is shown in Figure 4.

Before testing, the top and bottom surfaces of the specimen were polished to ensure flat contact with the inner surfaces of the steel bearing plates. A steel bearing plate was placed atop the specimen, allowing the vertical concentrated load to be uniformly distributed and applied to the top surface.

#### 2.3.2. Loading Protocol

According to Specification for Seismic Test of Buildings (JGJ/T 101-2015) [21], the preloading level was controlled within 30% of the estimated cracking load of the specimen to check the loading system, displacement transducers, load sensor, and strain acquisition system before the formal test. To ensure the flatness of the loading surface, each column specimen was subjected to three preloading cycles, each held for 5 min, prior to the formal test. During this process, the responses of the displacement transducers, load sensors, and strain gauges were checked for consistency with expected values. Additionally, the preloading served to compact any surface irregularities, thereby minimizing errors in the subsequent test. The formal test was conducted using a combined force-displacement graded loading protocol. In each loading step, a load corresponding to 10% of the peak load was applied and held for 2 min. When significant stiffness changes were observed in the load–displacement curve, the protocol switched to displacement-controlled loading at a rate of 0.2 mm/min. As the specimen approached its ultimate load, the loading rate was reduced to 0.1 mm/min and maintained until failure occurred.

#### 2.3.3. Arrangement of Sensor

**Figure 5:**
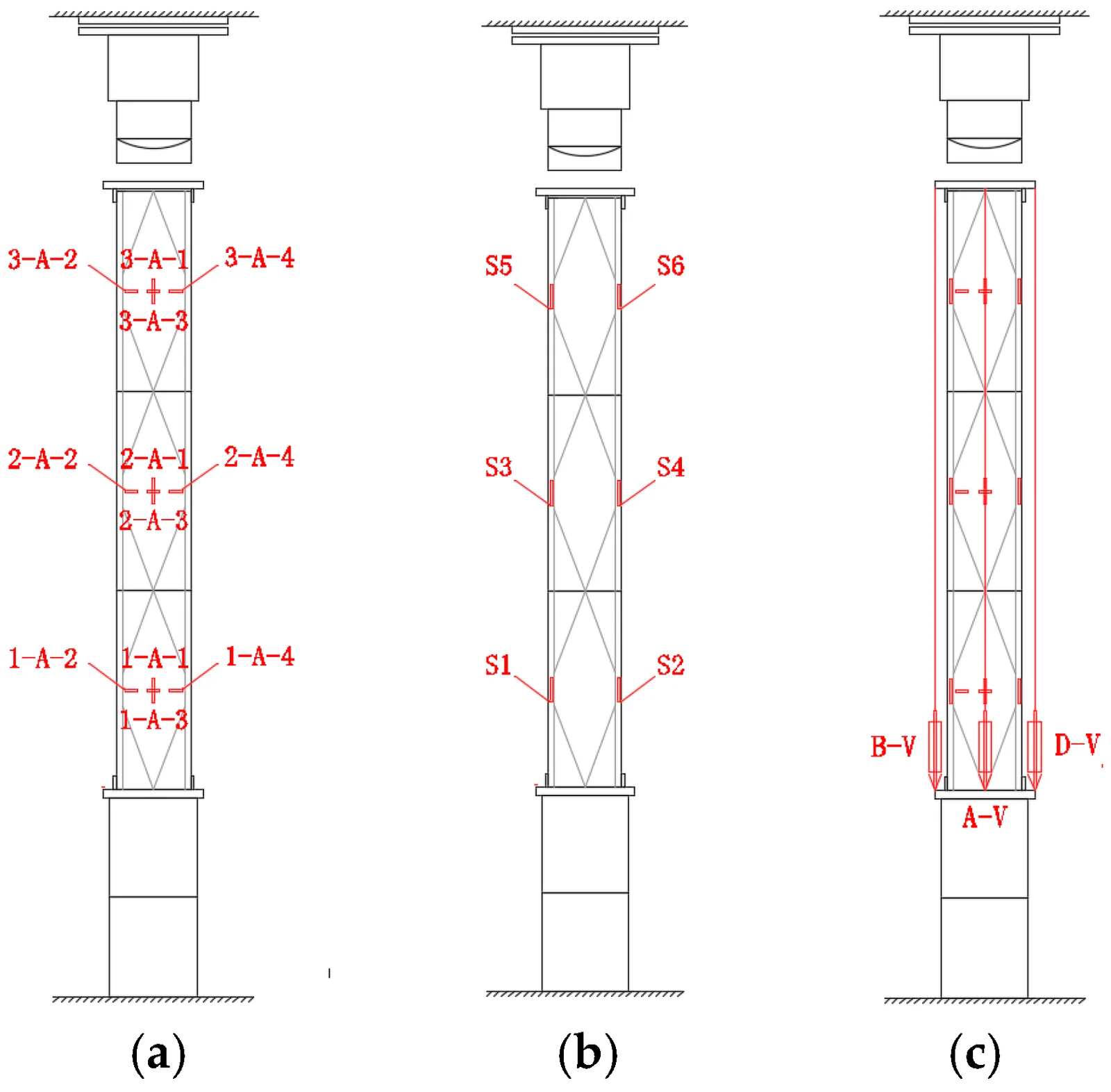
Layout of experimental measurement points. (**a**) Concrete strain; (**b**) aluminum strain; (**c**) displacement transducer locations.

To evaluate whether the aluminum alloy frame exerts a semi-passive confinement effect on the concrete, a total of 36 transverse concrete strain gauges were installed along the sides at the mid-height of each column segment, as shown in Figure 5a. Additionally, to investigate the failure locations and load transfer mechanism within each short column, 9 longitudinal concrete strain gauges were arranged at the mid-height, such as 1-A-1. Longitudinal strain gauges on the aluminum vertical bars (S1–S12) were arranged as illustrated in Figure 5b. Furthermore, four conventional displacement transducers were positioned on faces ABCD, as shown in Figure 5c.

The foam concrete strain gauges were long-gauge electrical resistance strain gauges with a gauge length of 60 mm, a resistance of 120 Ω, and a gauge factor of approximately 2.1. The aluminum alloy strain gauges were short-gauge electrical resistance strain gauges with a gauge length of 5 mm, a resistance of 120 Ω, and a gauge factor of approximately 2.1. This arrangement was adopted because long-gauge strain gauges are more suitable for measuring the average strain of heterogeneous cement-based materials, whereas short-gauge strain gauges are commonly used for metallic members with relatively uniform local deformation. Specifically, the foam concrete strain gauges were identified as TML PL-60-11-5LT long-gauge electrical resistance strain gauges (Tokyo Measuring Instruments Laboratory Co., Ltd., Tokyo, Japan), while the aluminum alloy strain gauges were identified as Kyowa KFG-5-120-C1-11L8M3R short-gauge electrical resistance strain gauges (Kyowa Electronic Instruments Co., Ltd., Tokyo, Japan). The displacement transducers used to measure the axial deformation were TML CDP-100 transducers (Tokyo Measuring Instruments Laboratory Co., Ltd., Tokyo, Japan).

## 3. Results and Analysis

### 3.1. Failure Modes

**Figure 6:**
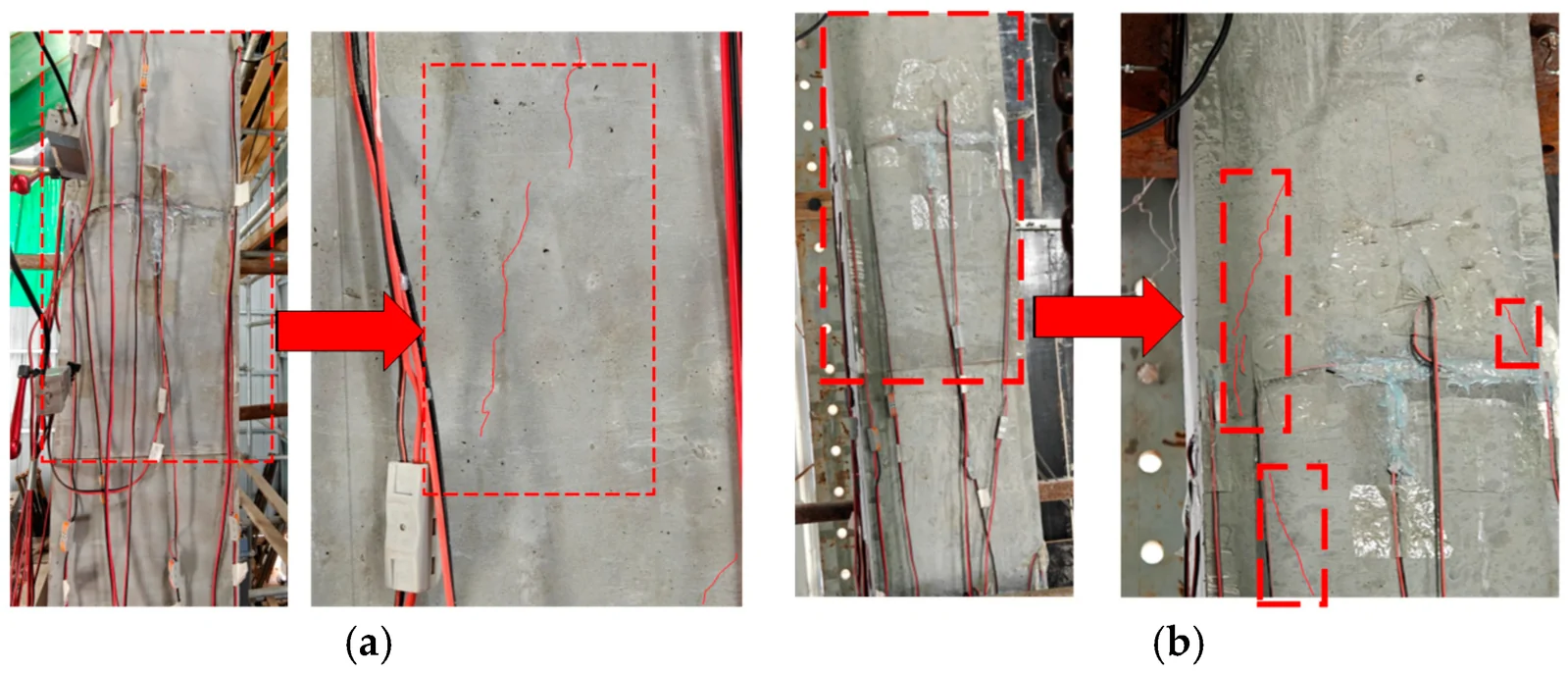
Failure patterns of the composite column test. (**a**) Failure mode of the middle column on face B; (**b**) failure mode of the middle column on face D.

In this study, one full-scale steel-skeleton composite joint specimen was tested under quasi-static cyclic loading. The first visible cracks were observed when the applied load reached 240 kN. It should be noted that, because no duplicate specimens with identical design parameters were tested, the repeatability of this cracking load cannot be statistically evaluated. Therefore, the cracking load and the observed failure sequence should be interpreted as specimen-level observations under the adopted geometry, material properties, axial compression ratio, and loading protocol, rather than as general statistical values for all joints of this type. Further repeated tests and parametric studies are needed to verify the dispersion and broader applicability of these results. When the axial load reached 240 kN, cracks began to appear in the concrete of the middle column on face B along the diamond-shaped diagonal braces, while a fine crack was observed on the surface of the middle column on face D, as shown in Figure 6.

Although only one full-scale specimen was tested in the present study, the observed failure pattern is consistent with the damage mechanisms reported in previous studies on foam concrete-filled or lightweight concrete-filled metal composite members. Xu [22] reported that foam concrete infill can restrain the cold-formed steel frame and change the failure mode of composite walls. Li [23] conducted axial compression tests on 12 thin-walled cold-formed steel tubular lightweight concrete columns and found that the typical failure mode involved local buckling of the steel tube and local crushing of the concrete. Previous studies on concrete-filled aluminum alloy tubular columns also showed that concrete infill can delay local buckling and improve the composite action between the aluminum alloy component and the concrete core. These findings support the interpretation that the cracking and damage concentration observed near the diagonal brace region in this study are related to local stiffness discontinuity, stress concentration, and load transfer between the aluminum alloy frame and foam concrete [24,25].

**Figure 7:**
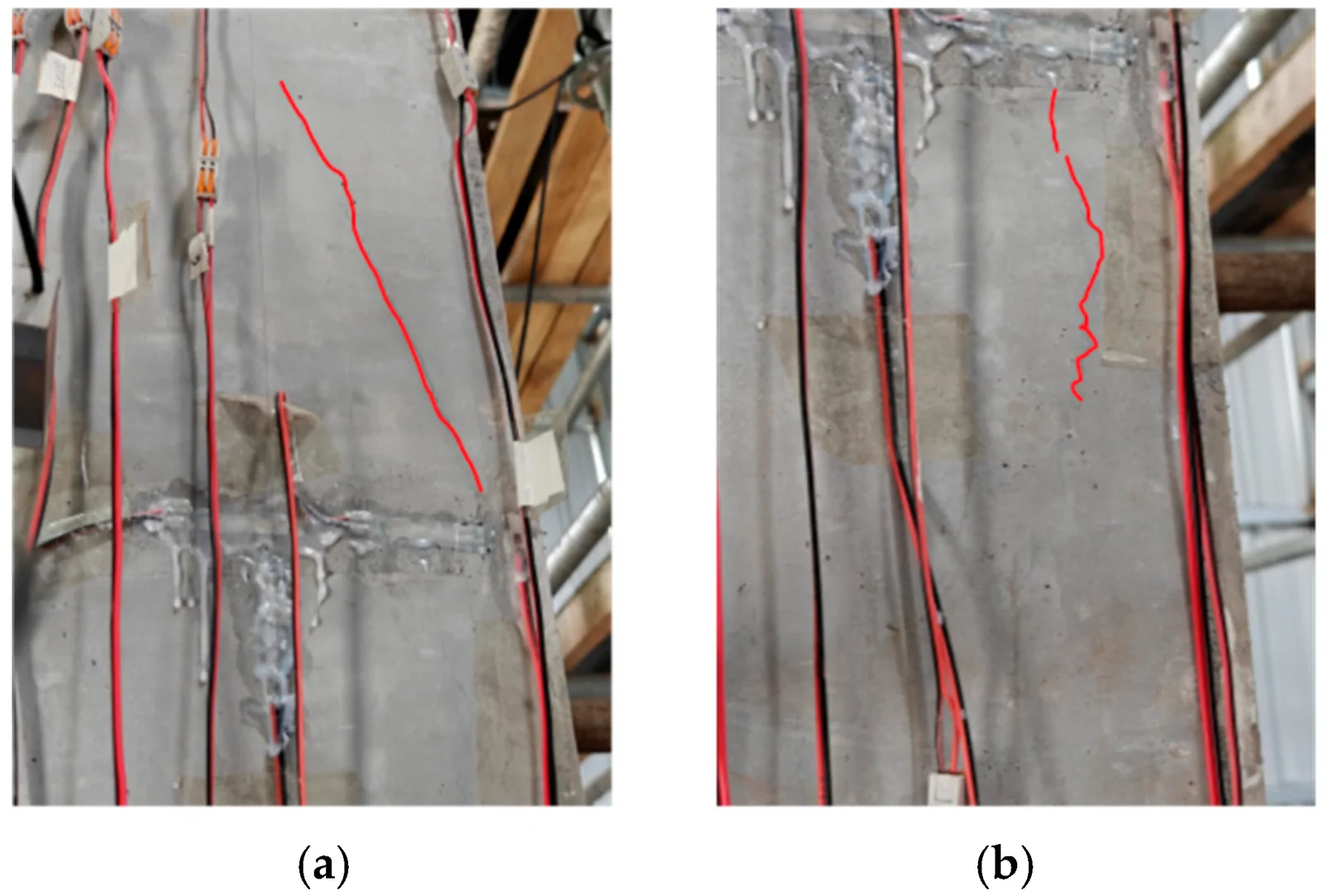
Failure pattern at an axial load of 300 kN. (**a**) The middle column on face D; (**b**) crack propagation. Note: The red lines in (**b**) mark the cracks observed on the foam concrete surface during the cracking process.

When the axial load increased to 330 kN, a concrete crack developed along the diagonal bracing direction in the mid-height region of the face D. The previously formed cracks further propagated, accompanied by a progressive increase in crack width, as shown in Figure 7.

**Figure 8:**
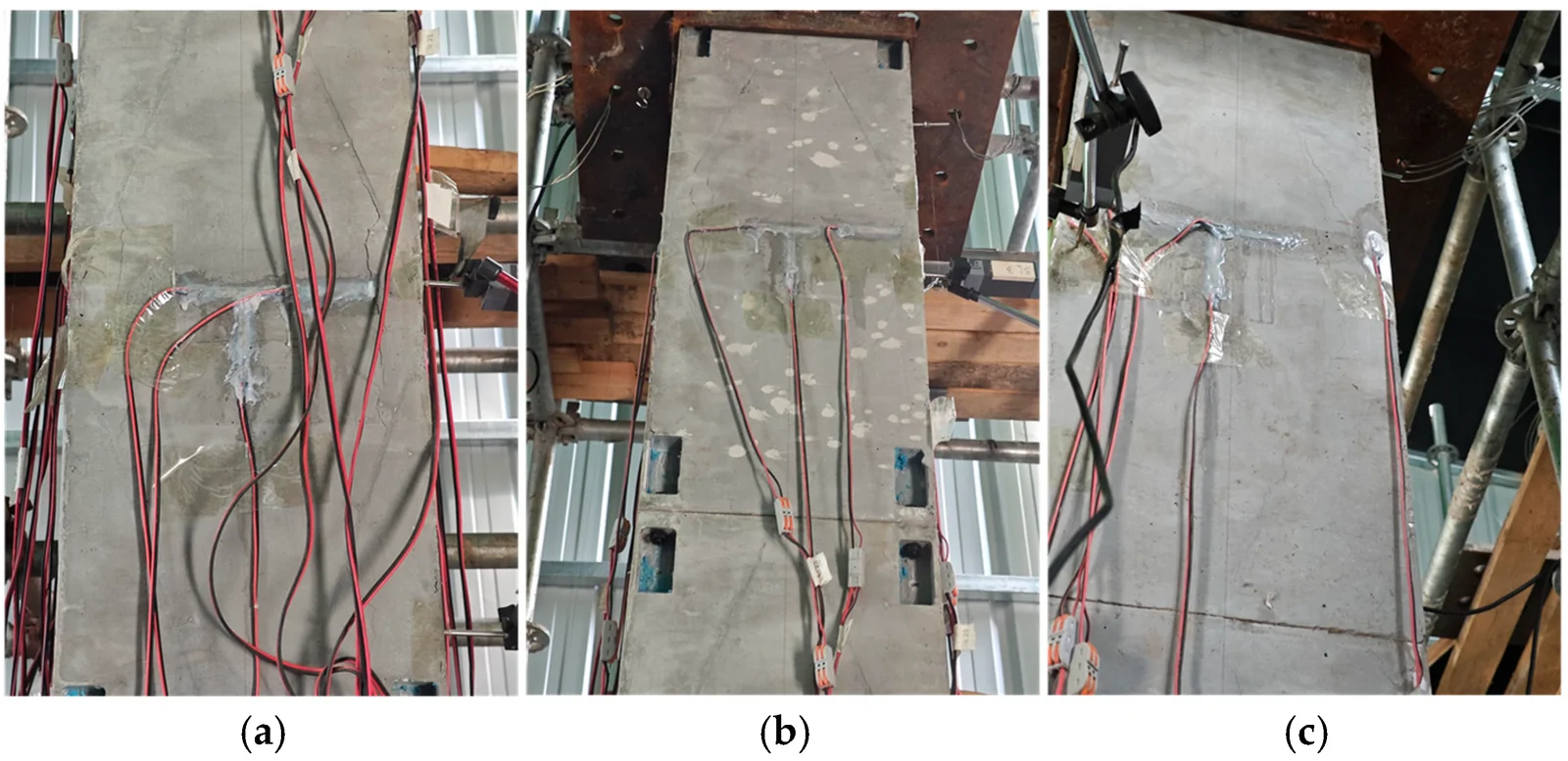

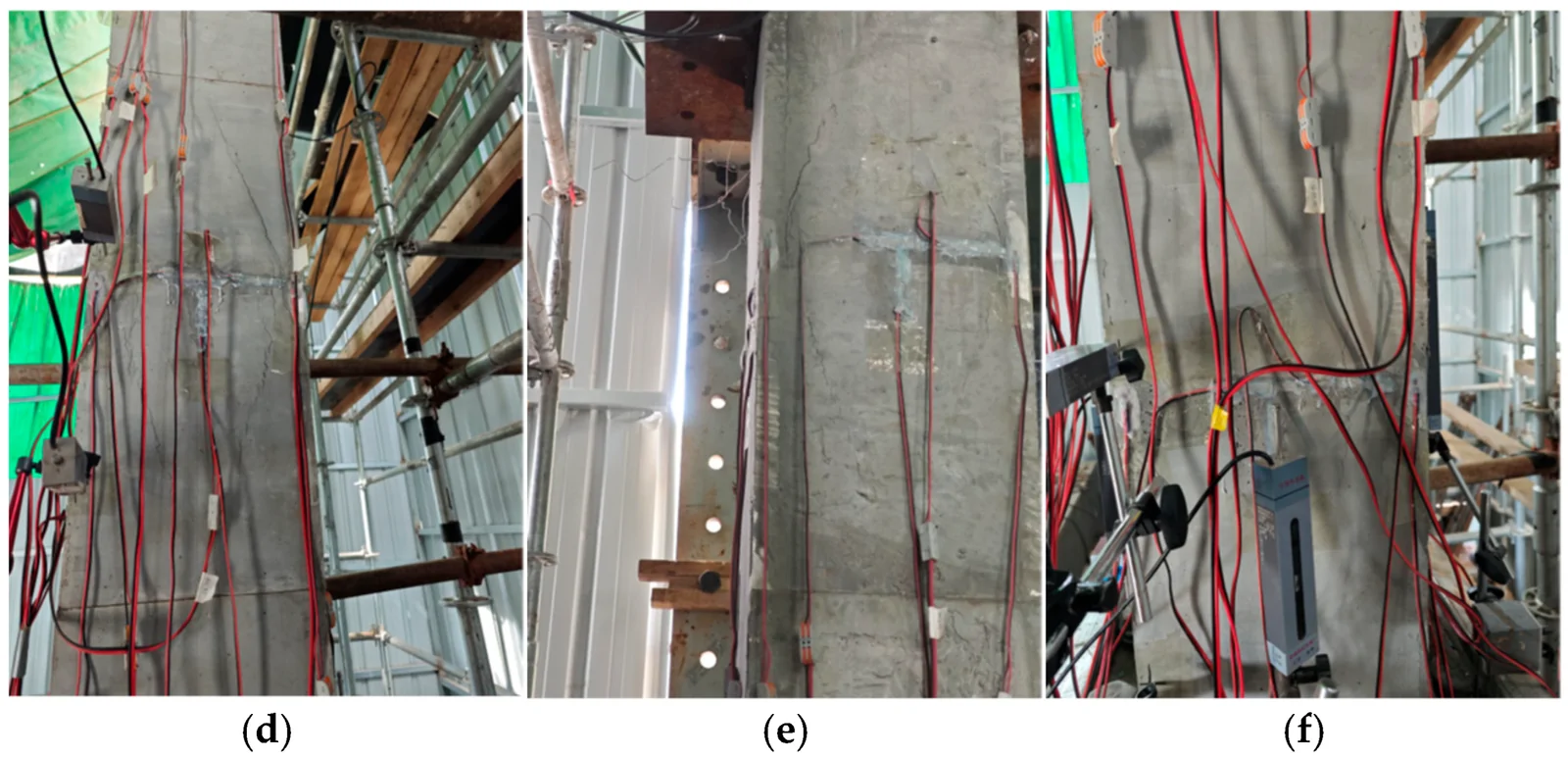
Damage evolution of the composite column under the 330–600 kN loading. (**a**) Central section of face A; (**b**) top section of the face A; (**c**) top section of the face A; (**d**) central section of face B; (**e**) top section of face B; (**f**) bottom section of face B.

As the load increased from 330 kN to 600 kN, the concrete cracks in the mid-height region of the BD faces progressively propagated along the diagonal bracing direction, accompanied by a continuous increase in crack width. Meanwhile, additional cracks also developed along the diagonal bracing direction and within the bracing zone on the ABD faces of both the top and bottom columns, as shown in Figure 8.

The crack distribution was not uniform on the four faces of the composite column. Cracks were mainly concentrated on faces B and D, where the diamond-shaped diagonal braces were closer to the concrete surface and caused more pronounced local stiffness discontinuity. In contrast, fewer visible cracks were observed on faces A and C. This difference indicates that the cracking behavior was closely related to the internal configuration of the aluminum alloy frame and the restraint condition provided by the diagonal braces.

The development of cracks along the direction of the diagonal braces can be attributed to the local stress concentration and nonuniform load transfer between the aluminum alloy frame and the foam concrete. Under axial compression, the vertical aluminum members carried a significant portion of the axial load, while the X-shaped diagonal braces provided lateral restraint and diagonal load-transfer paths. As the axial load increased, local deformation of the diagonal braces and relative interaction at the aluminum–concrete interface induced tensile and shear stresses in the surrounding foam concrete, leading to inclined cracking along the brace direction. Therefore, the observed cracking pattern reflects the combined effect of axial compression, local stiffness discontinuity, and interfacial load transfer.

**Figure 9:**
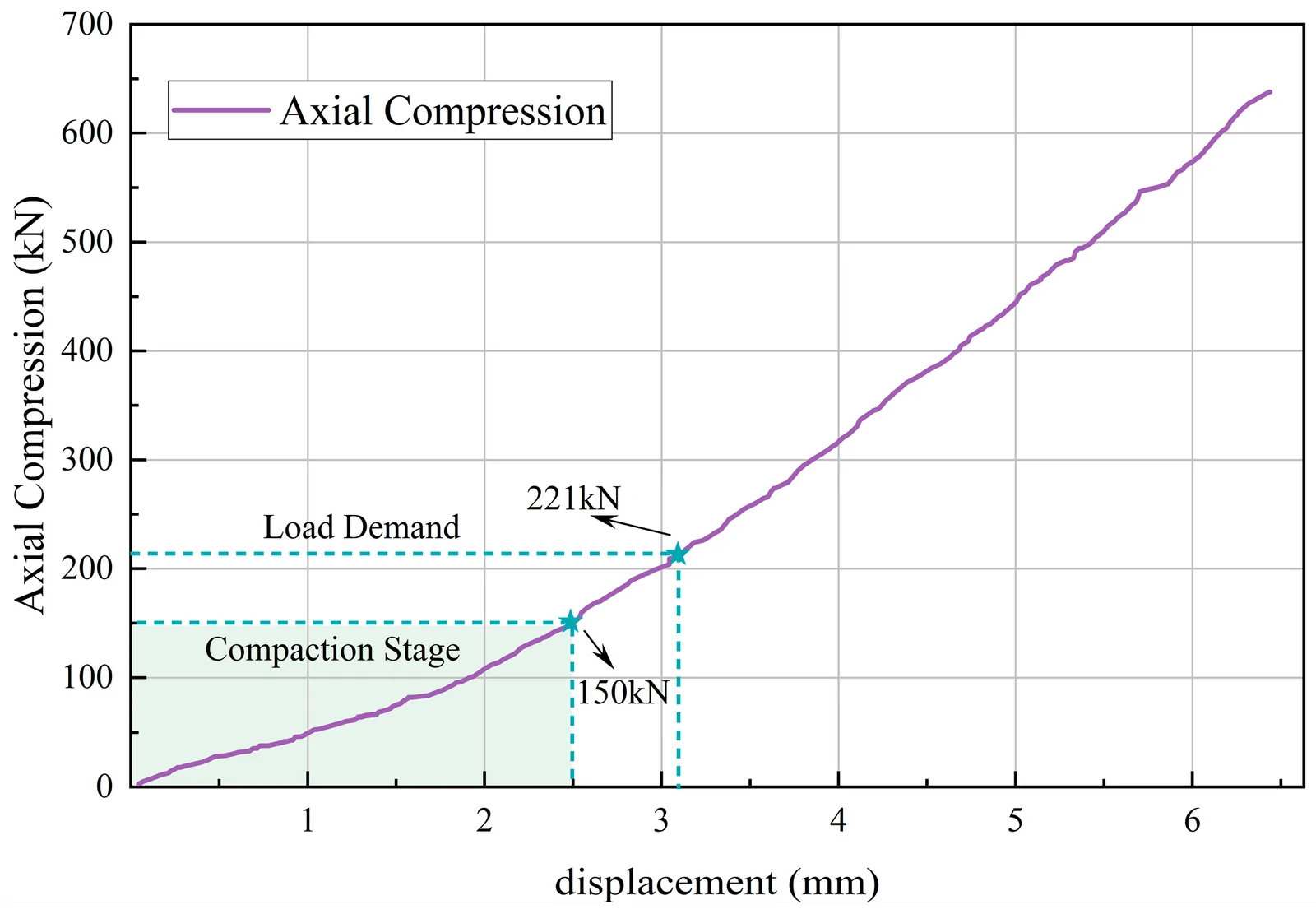
Axial load–displacement curve of the composite column.

When the applied load reached 240 kN, only minor cracks appeared in the concrete, indicating that the composite column still remained within the elastic range, satisfying the design requirements with a substantial safety margin. Based on the stiffness superposition principle, the elastic modulus of the concrete was calculated to be 900 MPa during the compaction stage and 2700 MPa during the elastic stage, as shown in Figure 9.

### 3.2. Stress–Strain Analysis of Composite Column

This section presents a systematic analysis of the axial load-strain behavior of the composite column specimens at different locations and along different orientations.

#### 3.2.1. Comparison of Transverse Strains

**Figure 10:**
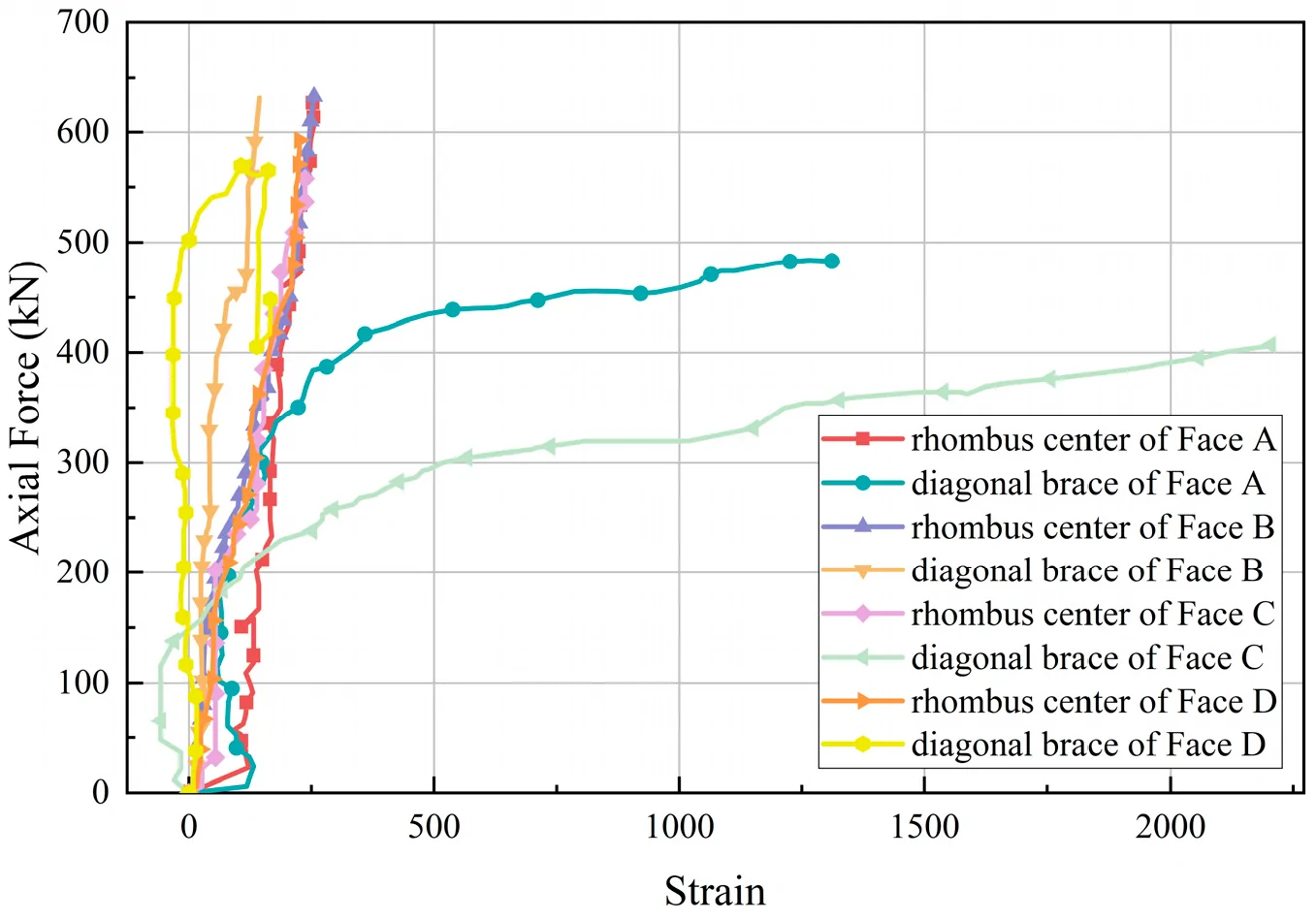
Bottom column transverse strain.

**Figure 11:**
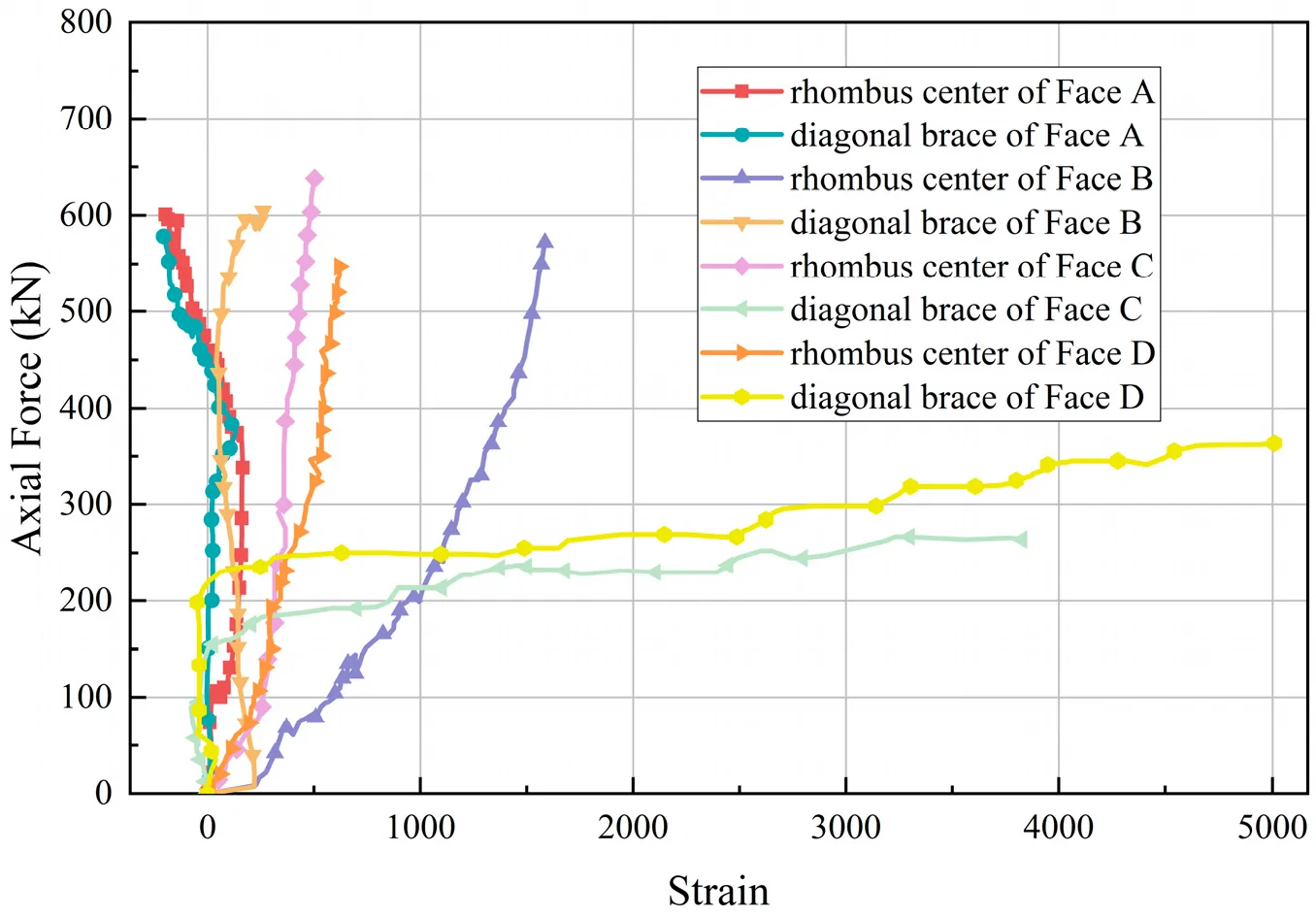
Middle column transverse strain.

**Figure 12:**
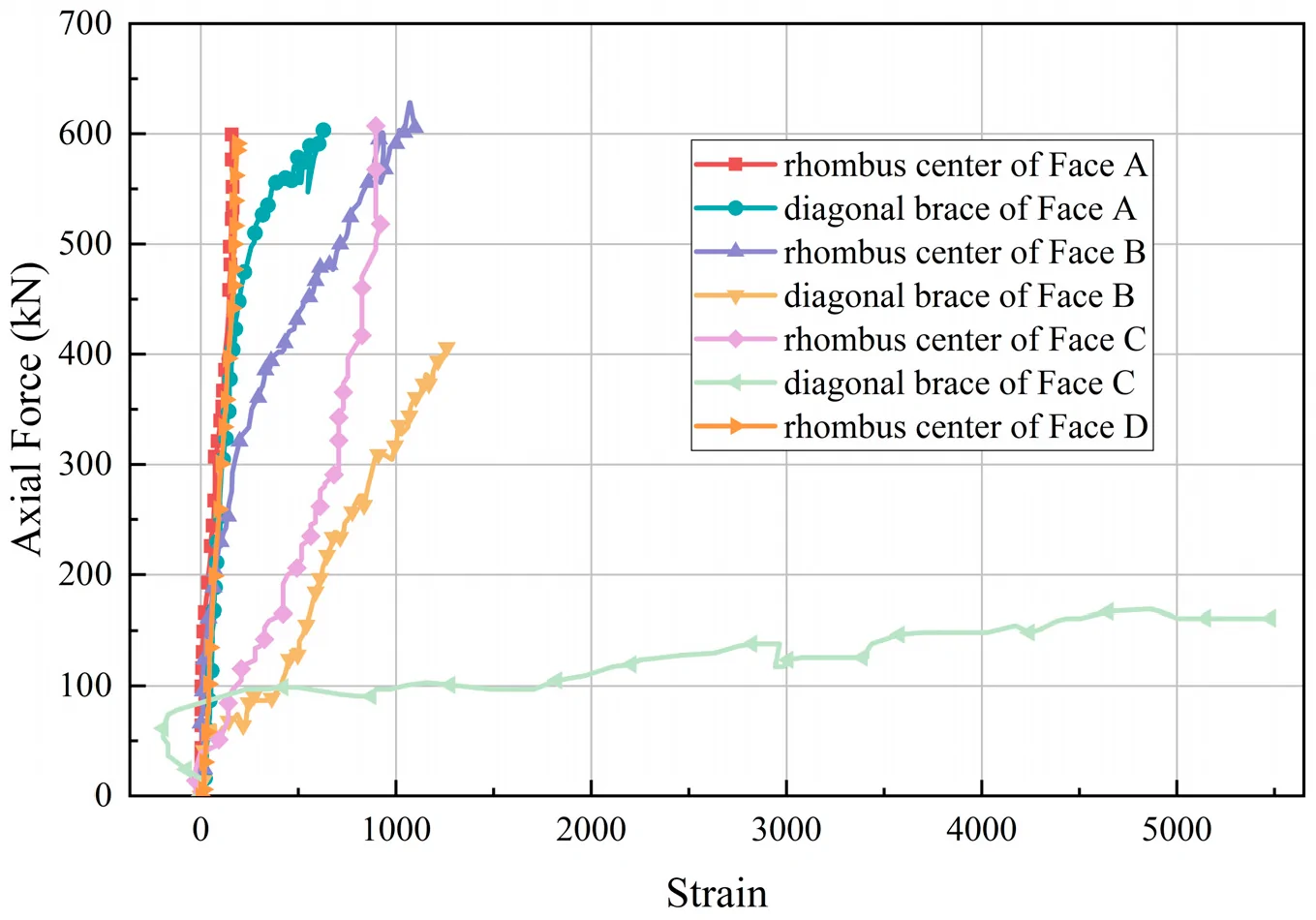
Top column transverse strain.

By comparing the transverse strains of the concrete at the rhombic welded reinforced regions and the horizontal mid-sections of each column segment, it was observed that the transverse deformation at the concrete core was consistently smaller than that at the peripheral concrete. The strain in the peripheral concrete exhibited an initial compression followed by expansion: the central concrete expanded under axial compression, producing positive strain, while the surrounding concrete experienced outward pressure from the core expansion combined with restraint from the aluminum vertical bars. During the initial loading stage, the peripheral concrete exhibited predominantly negative strains, indicating compression. As the load increased, the rhombic diagonal braces in the aluminum frame, acting as geometrically variable members, allowed slight lateral deformation of the vertical bars, thereby significantly reducing their semi-passive restraint effect on the surrounding concrete. The strain evolution trends are shown in Figure 10, Figure 11 and Figure 12.

#### 3.2.2. Comparison of Longitudinal Concrete Strains

**Figure 13:**
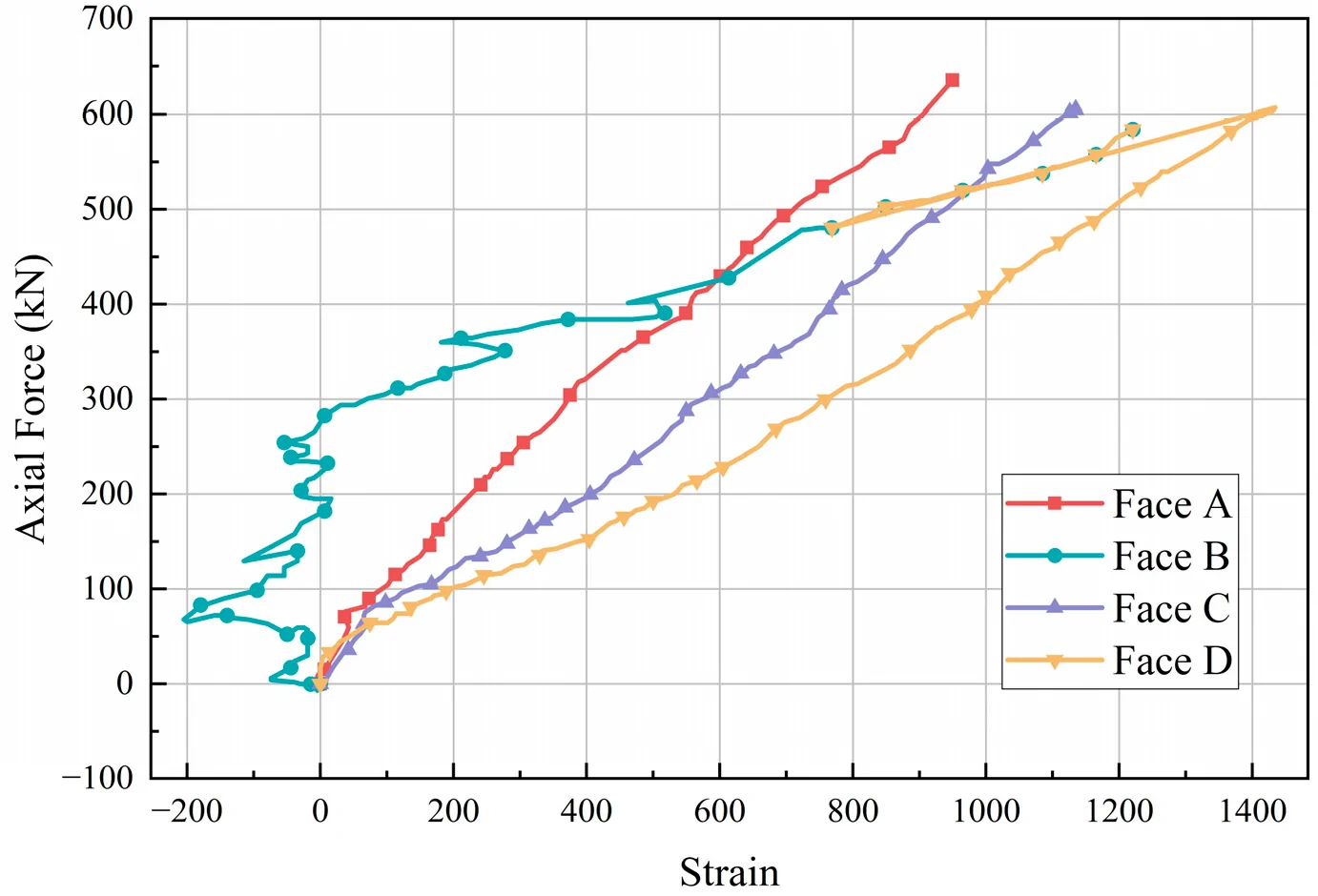
Strain curves of bottom column.

**Figure 14:**
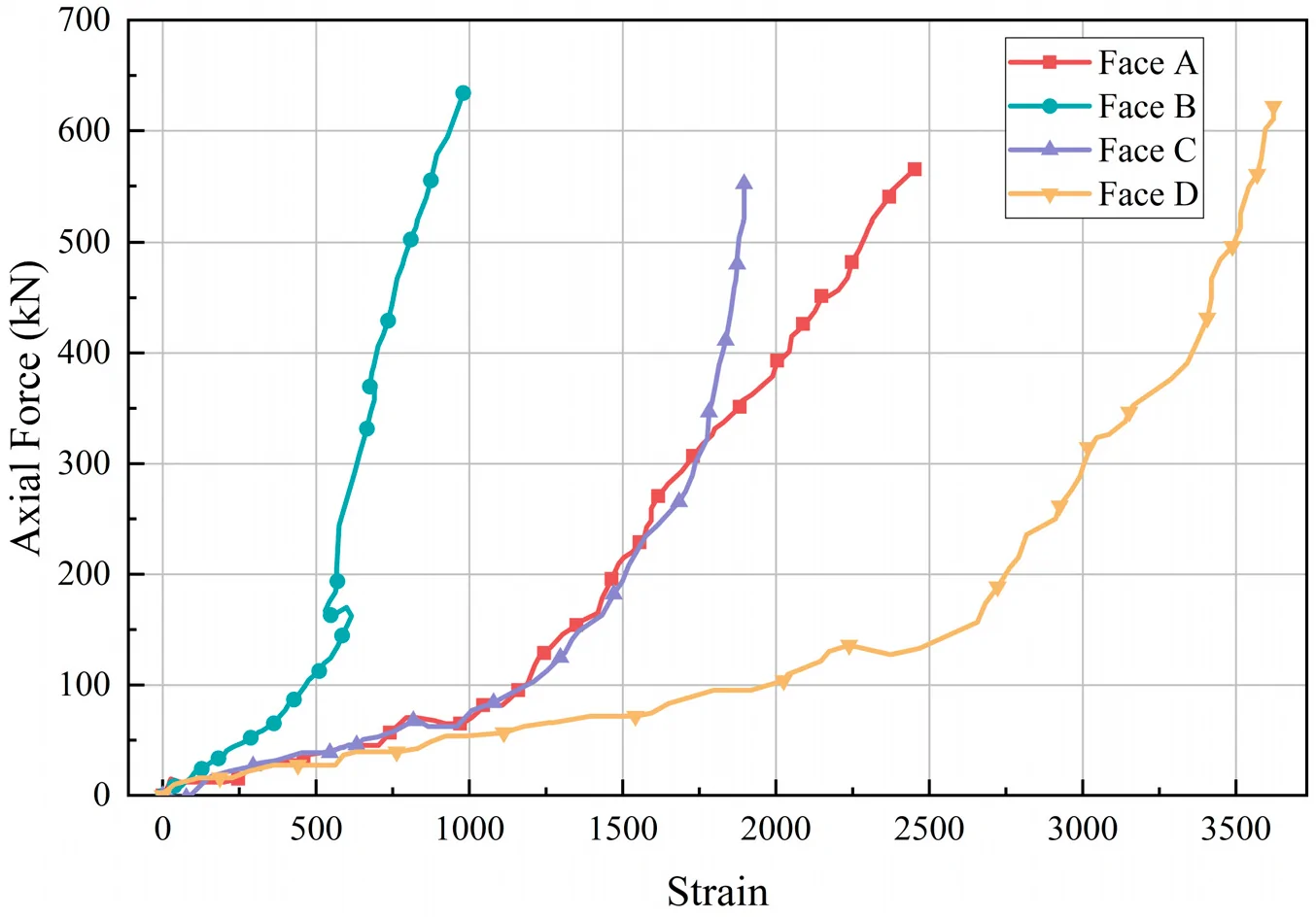
Strain curves of middle column.

**Figure 15:**
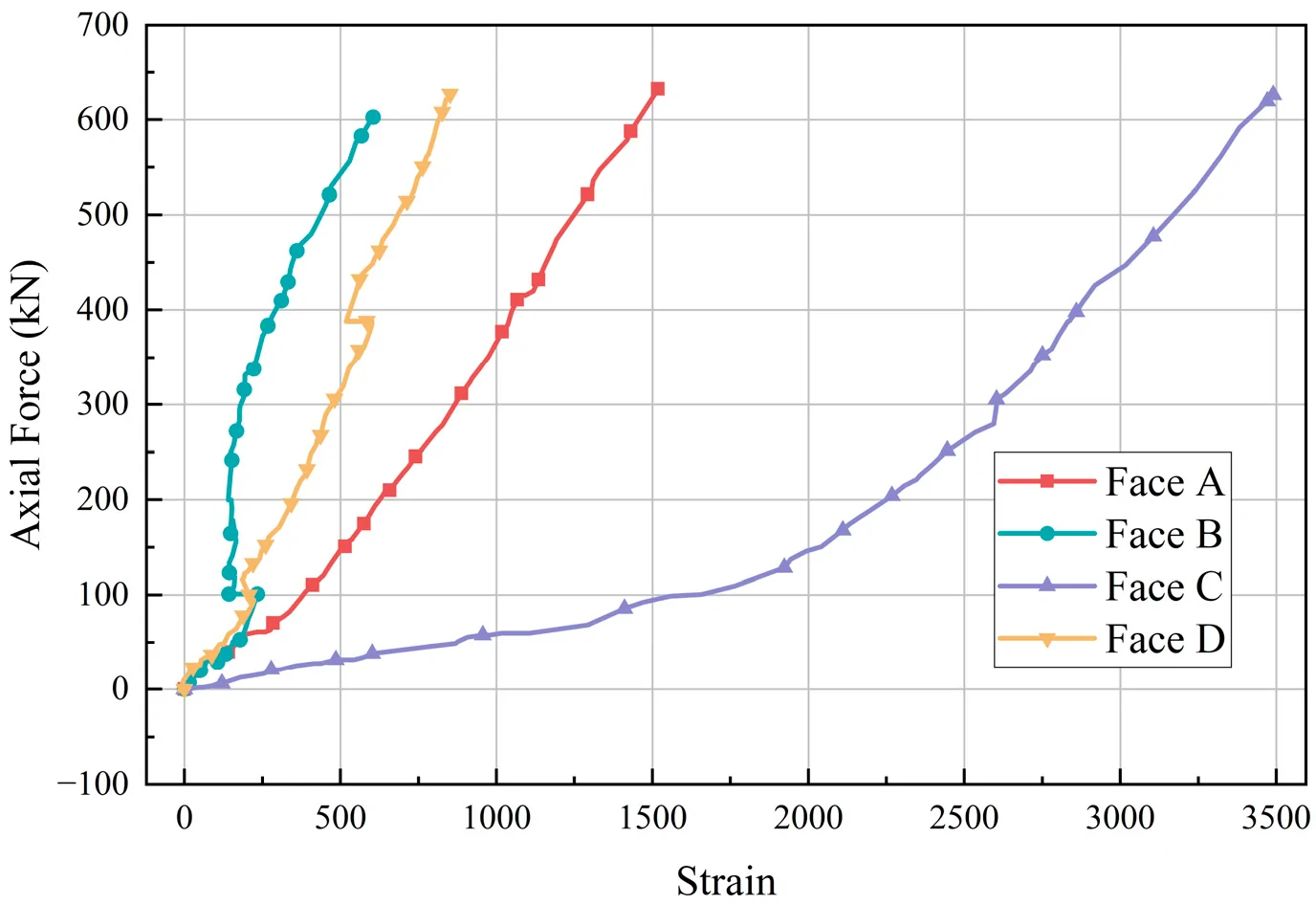
Strain curves of top column.

The longitudinal strain of concrete at the 1/6, 1/2, and 5/6 positions along the column height can be divided into two stages: the compaction stage and the elastic stage. The variation trend aligns with that of the axial load–displacement curve, indicating that during the initial loading phase, the foam concrete, as the primary compressive component, gradually increases in stiffness within a certain load range (approximately 1/10 to 1/15 of the peak load). The strain variation trends are shown in Figure 13, Figure 14 and Figure 15.

The division between the compaction stage and the elastic stage was determined according to the tangent stiffness variation in the load–strain curves. In the initial loading stage, the slopes of the load–strain curves changed noticeably, indicating the closure of initial pores, local bedding adjustment, and compaction of the foam concrete. When the load increased beyond this transition range, the load–strain curves became approximately linear, and the tangent stiffness remained relatively stable. This stage was therefore identified as the elastic working stage. According to the measured longitudinal strain curves, the compaction stage occurred mainly within the low-load range of approximately 40–60 kN for the bottom column, 60–100 kN for the middle column, and 50–100 kN for the top column. After these transition ranges, the strain responses at the 1/6, 1/2, and 5/6 heights increased almost linearly with the axial load, indicating that the foam concrete and aluminum alloy frame entered a more stable coordinated load-bearing state.

#### 3.2.3. Longitudinal Strain of Aluminum Alloy Frame

**Figure 16:**
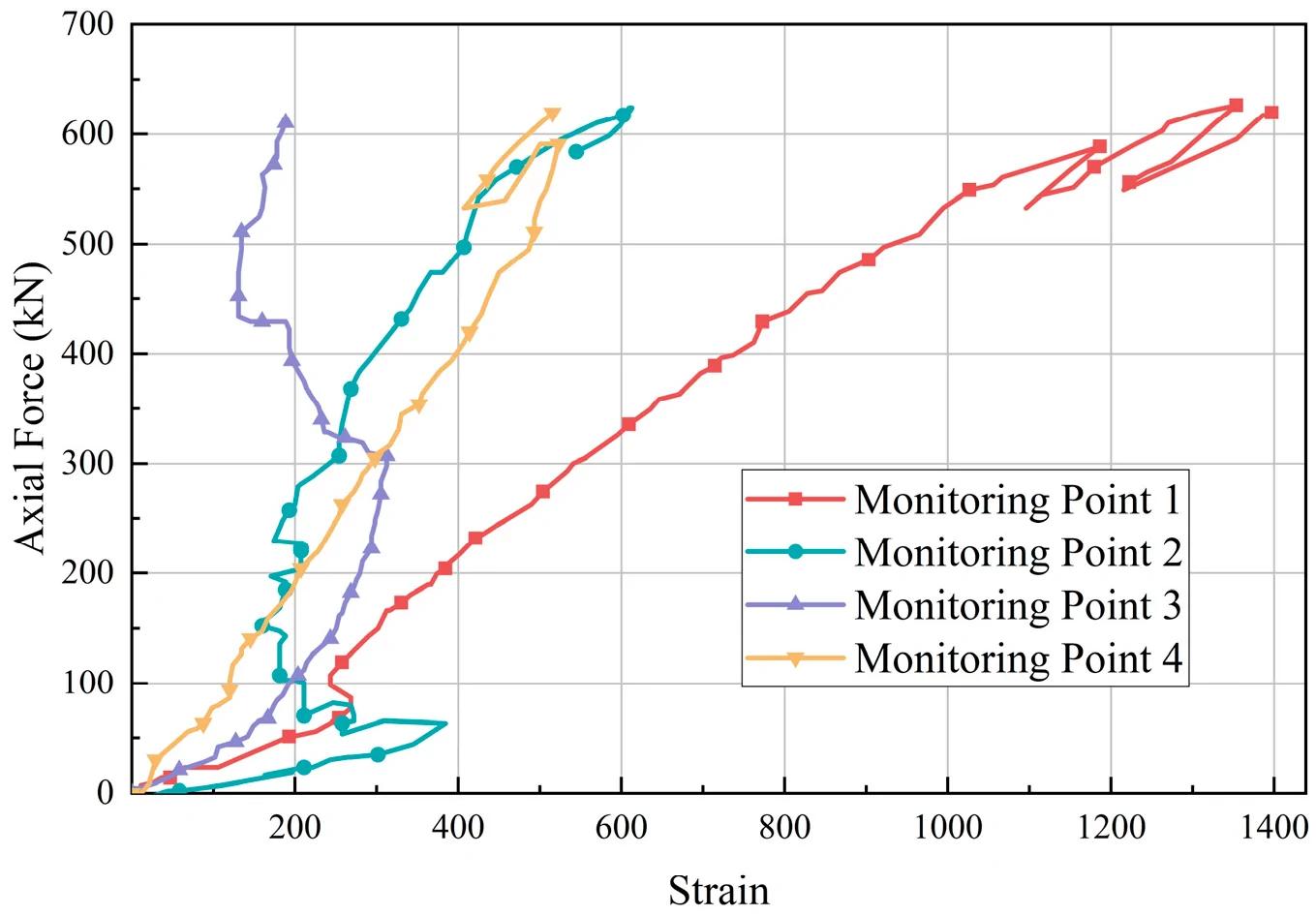
Longitudinal Strain of Aluminum Alloy frame in top Column.

**Figure 17:**
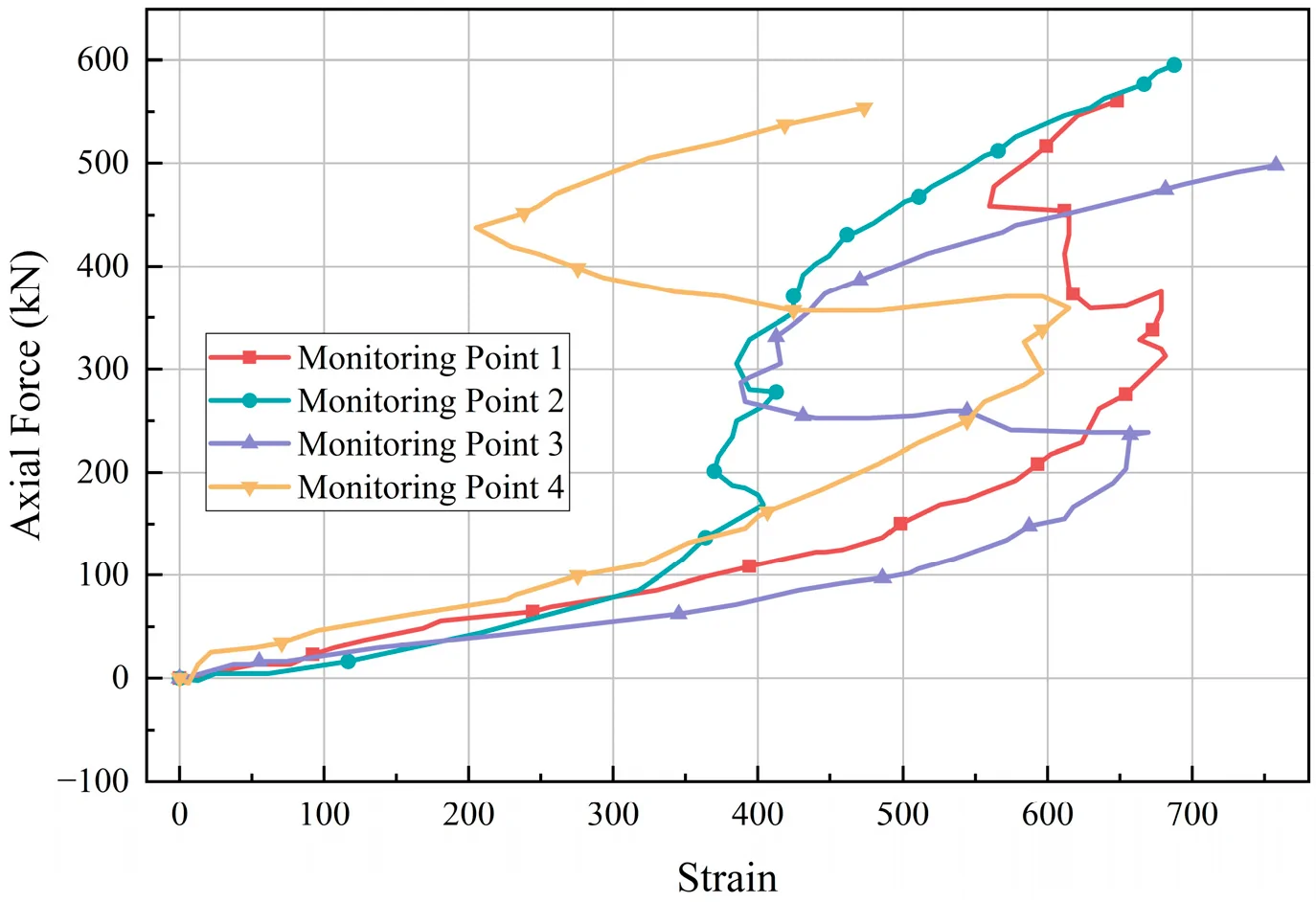
Longitudinal Strain of aluminum alloy frame in middle Column.

**Figure 18:**
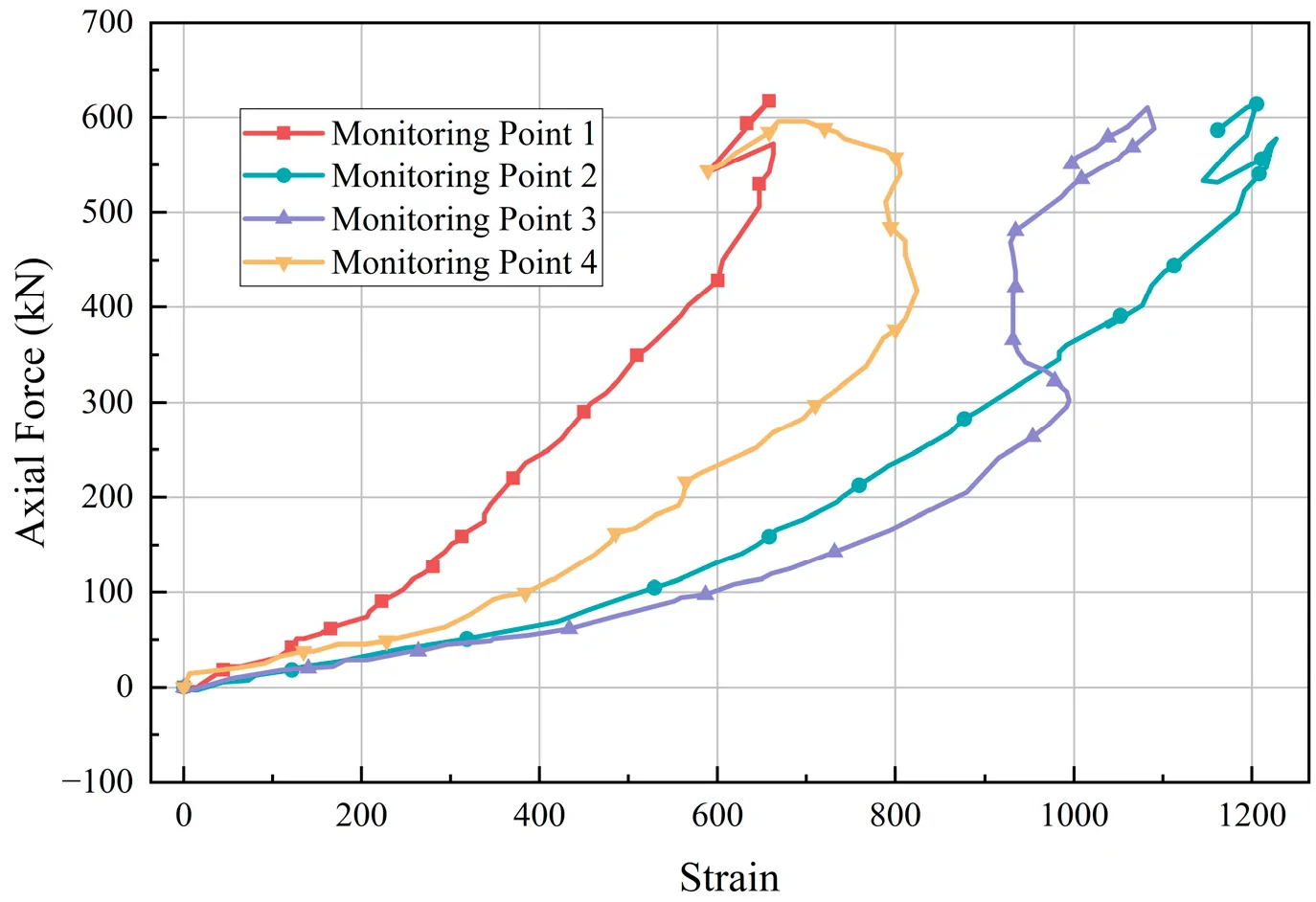
Longitudinal Strain of aluminum alloy frame in bottom Column.

At the initial loading stage, the aluminum alloy uprights similarly exhibit a progressive increase in stiffness. The corresponding strain variation trends are illustrated in Figure 16, Figure 17 and Figure 18. The longitudinal strain of the aluminum alloy uprights increased gradually with the axial load, indicating that the aluminum alloy frame progressively participated in axial load resistance during loading. In the initial loading stage, the strain growth rate was relatively small, which can be attributed to the adjustment of the contact state between the foam concrete and the aluminum alloy frame, as well as the compaction of the foam concrete. After this stage, the strain-load curves became more stable and approximately linear, suggesting that the aluminum alloy uprights entered a more effective load-bearing state.

Differences can also be observed among the top, middle, and bottom column segments. The middle column segment generally exhibited larger strain responses than the top and bottom segments, indicating that the middle segment carried a higher local load demand and was more sensitive to deformation concentration. This observation is consistent with the failure mode, in which cracks first appeared and developed more significantly in the middle column region. The strain development of the aluminum alloy uprights therefore confirms that the internal frame contributed to axial load transfer and influenced the damage distribution of the composite column.

## 4. Finite Element Modeling

Using FE simulation software ABAQUS 6.14, in this section, the FEM of composite columns is established and then employed to study the stress distribution of concrete and aluminum alloy frame, as well as the interaction mechanism between concrete and aluminum alloy frame.

### 4.1. Finite Element Model Construction

**Figure 19:**
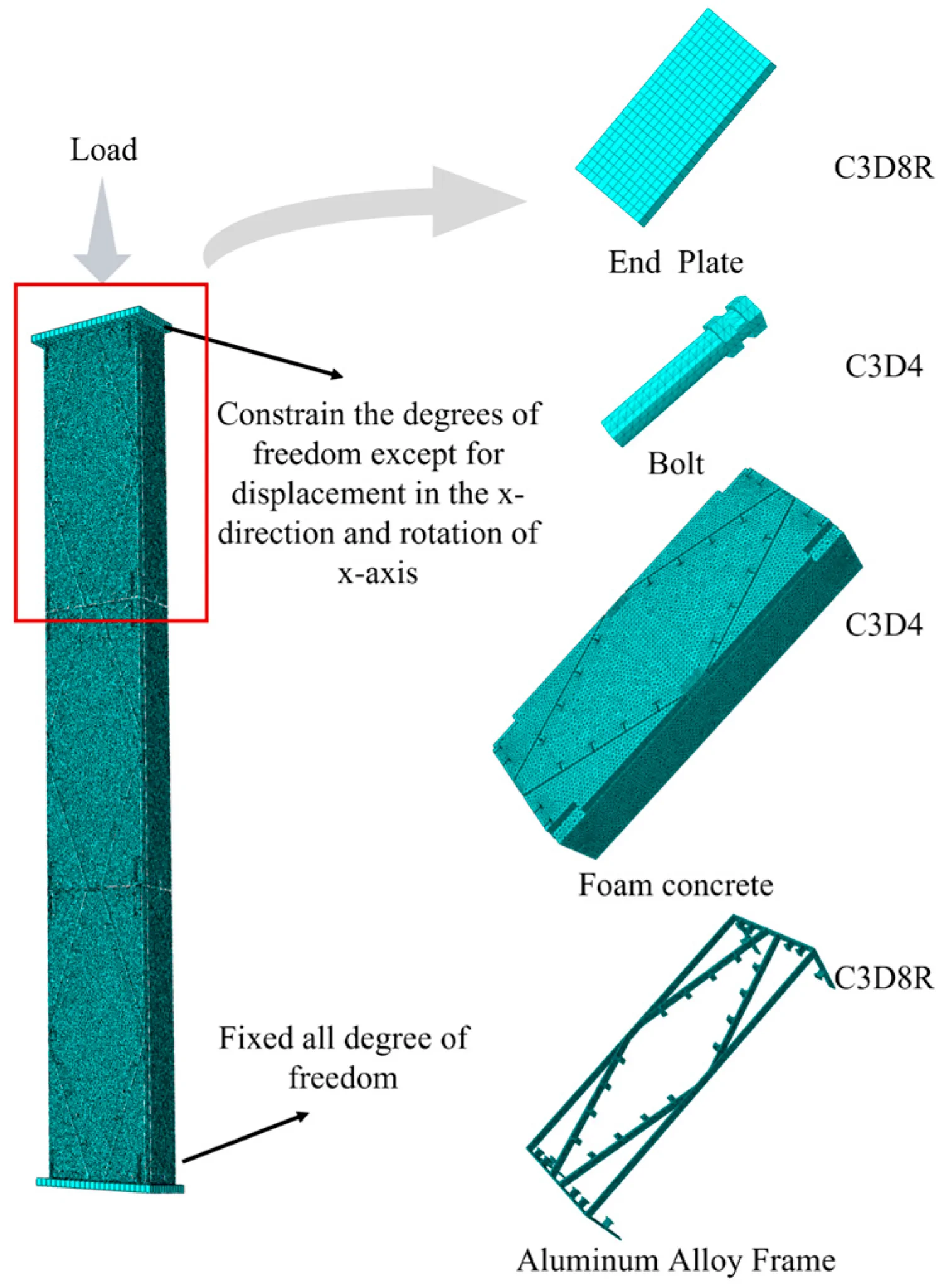
FEM of composite column. Note: C3D8R elements for the loading plates and aluminum alloy frame, and C3D4 elements for the bolts and foam concrete.

Based on the configurations shown in Figure 1 and Figure 4, a FEM was established to perform numerical simulations under the same loading conditions. A half-model was adopted, and symmetric boundary conditions were applied at the cutting plane, as shown in Figure 19.

### 4.2. Material Modeling

#### 4.2.1. Modelling of Aluminum Alloy Frame

Under multiaxial stress conditions, the Mises yield criterion and the associated plastic flow rule were adopted. The aluminum alloy was assumed to exhibit isotropic hardening behavior. ABAQUS provides material characterization using the PLASTIC option, which enables the definition of a multilinear stress–strain curve [26]. The initial segment of the multilinear curve represents the elastic response, extending to the proportional limit corresponding to the measured Young’s modulus and a Poisson’s ratio of 0.33. The material properties used for the aluminum alloy frame are summarized in Table 3.

#### 4.2.2. Foam Concrete

The Concrete Damage Plasticity (CDP) model [27] can simulate a wide range of quasi-brittle materials. In this study, the triaxial plastic damage constitutive model for concrete under axial compression is employed. The uniaxial stress–strain relationship of the infill concrete can be expressed as:

(1) $$y=\left\{\begin{array}{c} \frac{Ax+(B-1)x^{2}}{1+(A-2)x+Bx^{2}},x\leq 1\\ \frac{x}{\alpha {\left(x-1\right)}^{2}+x},x>1 \end{array}\right.$$

*y* = *σ*/*fc* and *x* = *ε*/*ε_c_* represent the normalized stress and strain of the infill concrete under uniaxial compression, respectively. *σ* and *ε* denote the stress and strain of the infill concrete. *f_c_* = 0.4*f_cu_*^7/6^ is the uniaxial compressive strength of the concrete, where *f*_cu_ represents the cube compressive strength. *ε_c_* is the strain corresponding to the peak compressive stress of the concrete, given by *ε_c_* = 383*f_cu_*^7/18^ × 10^−6^. *A* is the ratio of the initial tangent modulus to the secant modulus at the peak stress, expressed as *A* = 9.1*f_cu_*^−4/9^. *B* = 1.6 (*A* − 1)^2^ is a parameter controlling the reduction in the elastic modulus along the ascending branch of the axial stress–strain curve. In general, the parameter *α* is taken as 0.15 [28].

The triaxial plastic damage behavior of the infill concrete is defined using the CDP model, with an eccentricity of 0.1, a ratio of the initial biaxial compressive yield stress to the initial uniaxial compressive yield stress (*f*_b0_/*f*_c0_) of 1.225, a ratio of the second stress invariant on the tensile meridian to that on the compressive meridian of 2/3, a viscosity parameter of 0.005, and a dilation angle of 40°. This constitutive model is independent of the shape and material of the metal tube infill, making it suitable for simulating the axial compression behavior of aluminum alloy–lightweight concrete short columns.

### 4.3. Element Mesh, Contact Type, Loading Method and Boundary Condition

The aluminum alloy frame was modeled using C3D8R solid elements, while the foam concrete was simulated using C3D4 elements. To ensure both numerical accuracy and computational efficiency, a structured meshing technique available in ABAQUS was adopted, and a mesh convergence study was performed. Following the recommendation of Zhou and Young [29], the element aspect ratio was maintained as close as possible to 1:1:1. In this study, the mesh size for the concrete elements was set to 10 mm using tetrahedral elements. For the aluminum alloy frame, hexahedral elements were employed with a global mesh size of 5 mm. The typical mesh configuration of the entire model is shown in Figure 19.

### 4.4. Verification of FE Model

**Figure 20:**
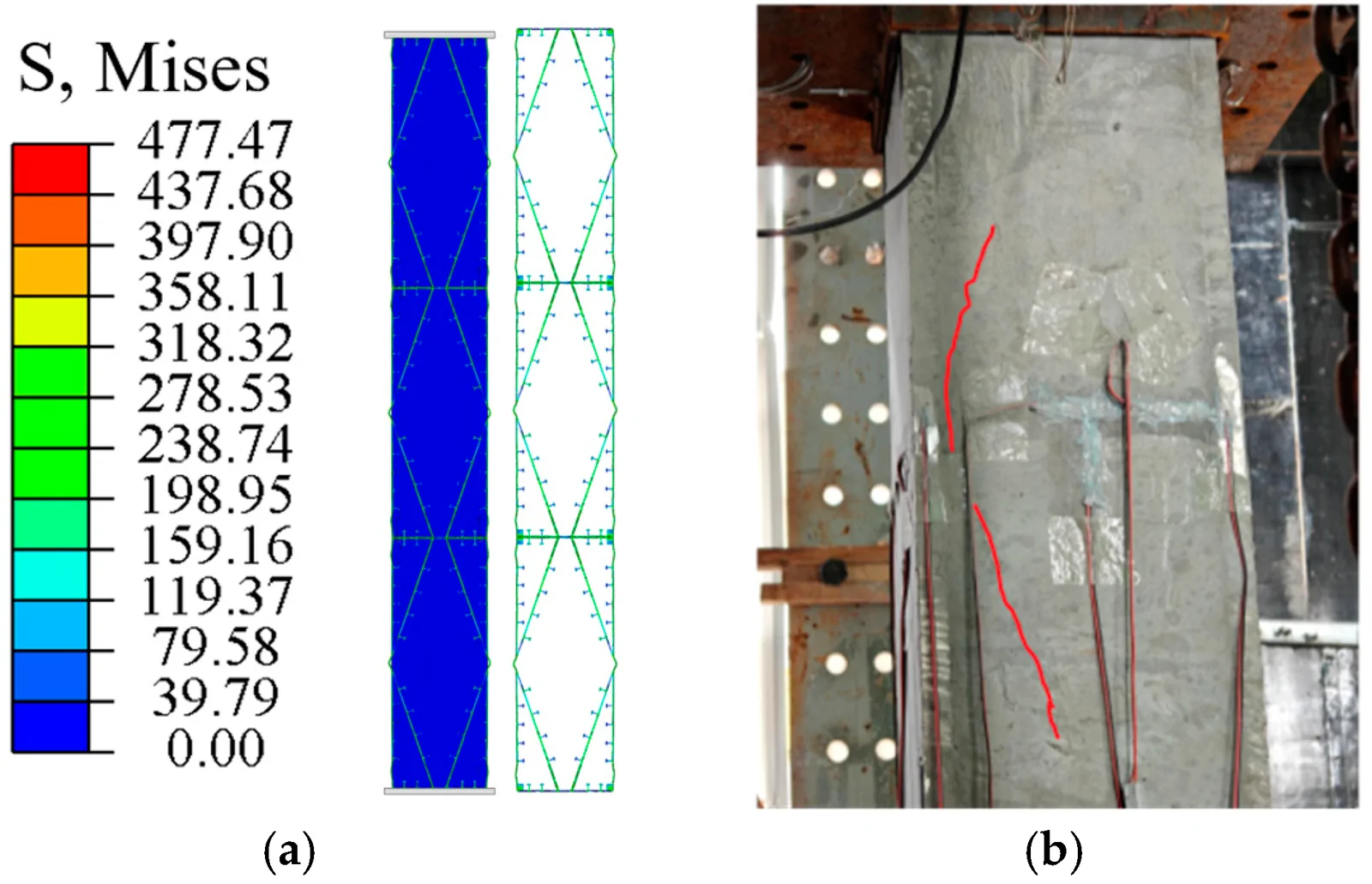
Comparison of failure modes. (**a**) FE failure mode; (**b**) experimental failure mode. Note: The red lines in (**b**) mark the cracks observed on the foam concrete surface during the cracking process.

To ensure the validity of the numerical simulation, axial compression tests were conducted on existing composite columns. A comparison of the failure modes obtained from the experiments and finite element analyses is presented in Figure 20. The FEM predicts high stress concentrations in the diagonal web region of the aluminum alloy frame, which corresponds well with the diagonal cracking observed in the experiments, demonstrating good agreement between the numerical and experimental failure characteristics. The correlation between the two images is based on the consistency between the stress/damage concentration regions predicted by the finite element model and the spatial location and development direction of the inclined cracks observed in the experiment. Specifically, the finite element model showed stress concentration near the diagonal brace regions of the aluminum alloy frame, while the experimental specimen exhibited inclined cracks in the surrounding foam concrete along the corresponding brace direction. Therefore, the two images are correlated through the agreement between the predicted damage-prone regions and the actual crack paths observed in the test.

At present, no existing design code provides a direct calculation method for aluminum alloy–foam concrete composite columns with embedded spatial frames, diagonal braces, interlocking keys, and segmented bolted connections. Nevertheless, to provide an approximate engineering comparison, a simplified equivalent-area superposition method was adopted in this study. The equivalent axial area of each material was calculated from the corresponding material volume divided by the specimen height:

$$N_{u}=f_{a}A_{a,eq}+f_{c}A_{c,eq}$$

where $A_{a,eq}$ and $A_{c,eq}$ are the equivalent axial areas of the aluminum alloy frame and foam concrete, respectively; $f_{a}$ is the 0.2% proof strength of the aluminum alloy; and $f_{c}$ is the measured compressive strength of the foam concrete. This calculation does not include the beneficial effects of interlocking keys, confinement, interface interaction, or load redistribution. Therefore, the calculated value should be regarded as a conservative approximate reference rather than a direct design-code prediction. The comparison results are presented in Table 4.

**Figure 21:**
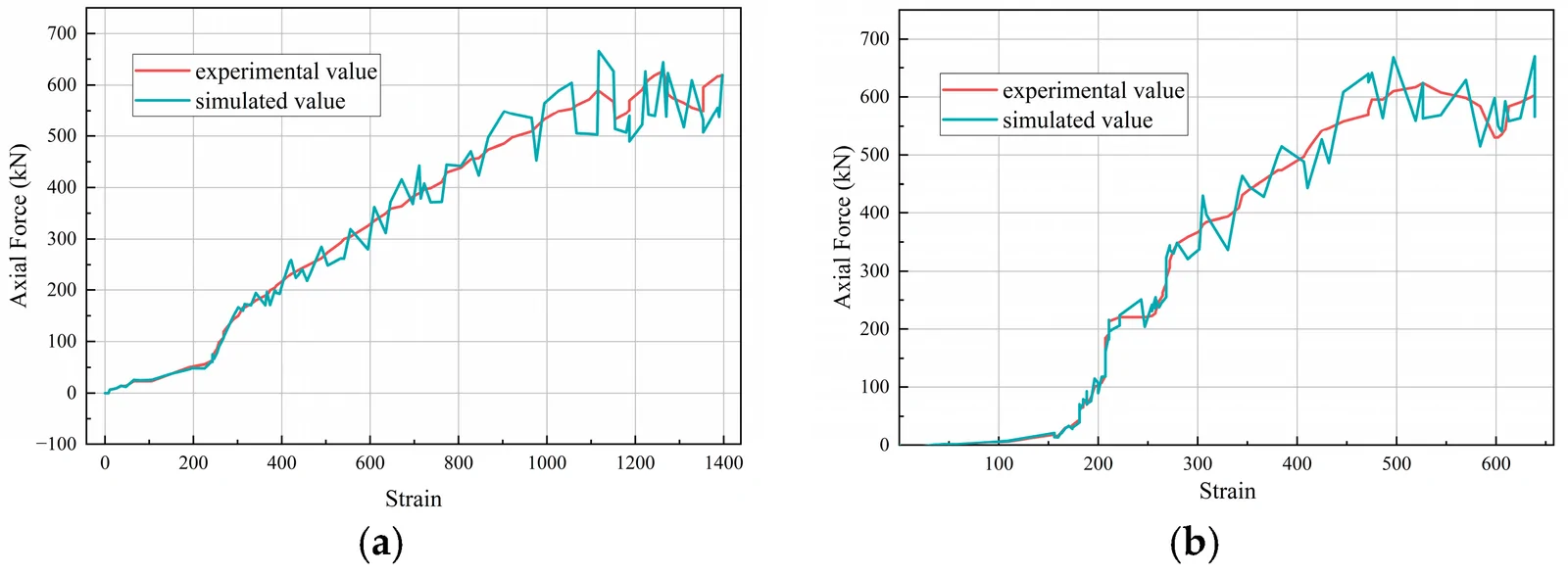
Comparison of strains in the aluminum alloy frame. (**a**) Diagonal web of the top column; (**b**) middle vertical member of top column.

The strain curves of top column presents that the overall strain trends are generally consistent between the two cases, as shown in Figure 21.

Local fluctuations can be observed in the simulated axial force response in Figure 21. This phenomenon is mainly related to the nonlinear damage evolution and load redistribution of the composite column during loading. In the finite element analysis, the foam concrete was modeled using the CDP model. As the axial load increased, local compressive damage, plastic strain development, and stiffness degradation occurred in the foam concrete, especially near the diagonal brace regions where stress concentration was more pronounced. The development of local damage changed the tangent stiffness of the model and caused redistribution of axial force between the aluminum alloy frame and the surrounding foam concrete. Therefore, short fluctuations in the simulated axial force curve can be interpreted as the numerical response associated with local damage evolution, stiffness degradation, and load redistribution in the composite member. Although local fluctuations are present, the simulated curve captures the overall trend and peak load of the experimental response, indicating that the model has acceptable accuracy for subsequent parametric analysis.

To quantitatively evaluate the accuracy of the finite element model, the peak load deviation, and mean relative error were calculated by comparing the experimental and numerical load–displacement and representative strain responses, as shown in Table 5. The peak load deviation was calculated as the absolute difference between the experimental and numerical peak loads divided by the experimental peak load.

## 5. Parametric Study

Based on the finite element model validated against the full-scale axial compression test, a numerical parametric study was conducted to investigate the effects of cover thickness, number of interlocking keys, and aluminum alloy frame thickness on the axial compression capacity of the composite column. It should be noted that these parameter combinations were evaluated numerically, and no additional physical tests were conducted for each parameter case.

### 5.1. Cover Thicknesses

**Figure 22:**
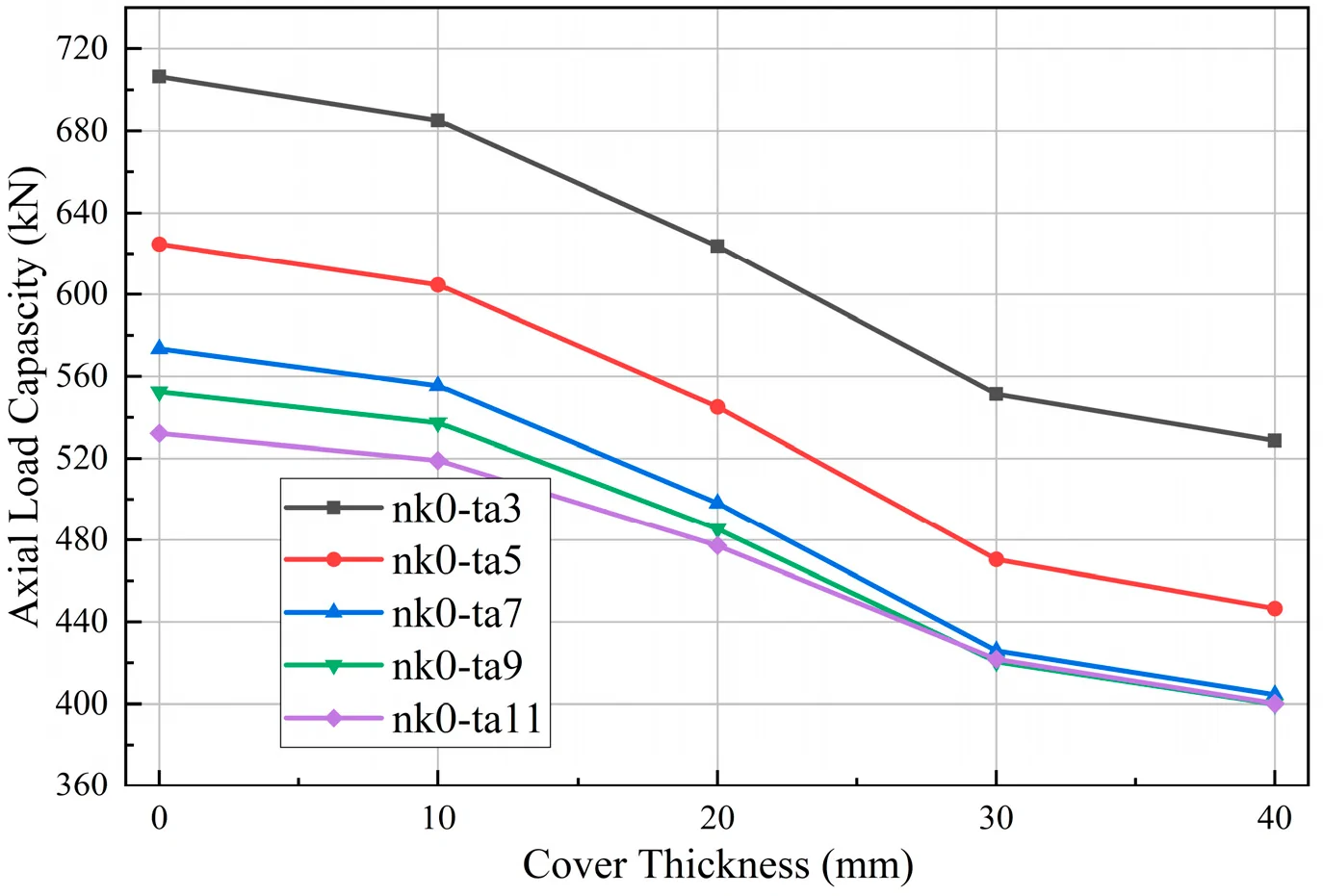
Influence of the Cover thickness.

The influence of the concrete cover thickness on the axial compressive capacity of the composite column is shown in Figure 22. The results indicate that the axial capacity decreases progressively with increasing cover thickness. This reduction is attributed to the corresponding decrease in the volume of the aluminum alloy frame as the cover becomes thicker. Since the aluminum alloy frame serves as the primary load-bearing component, its reduction in size leads directly to a decline in the overall axial compressive capacity.

### 5.2. Interlocking Key Number

**Figure 23:**
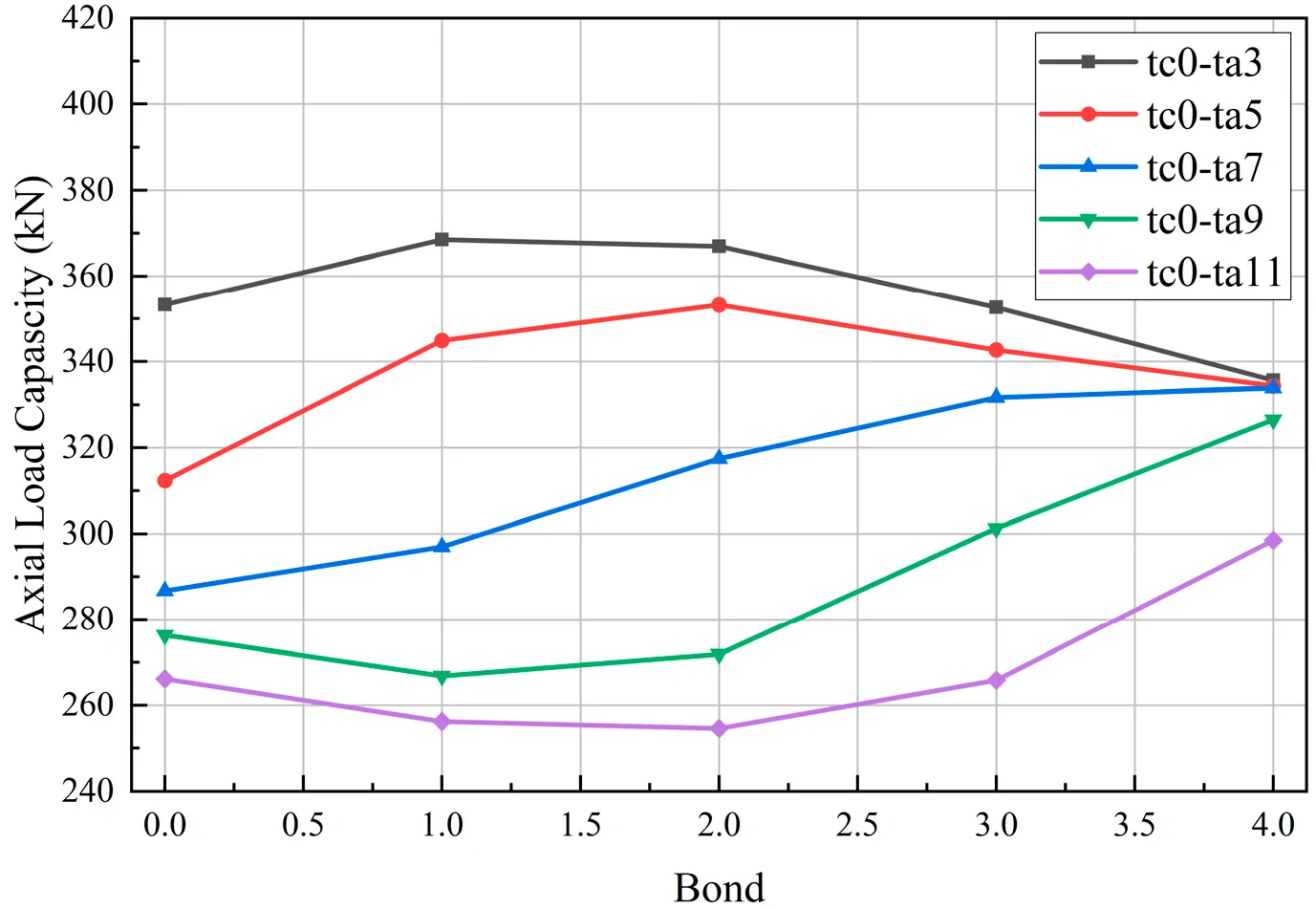
Influence of the Interlocking key number.

The influence of the interlocking key on the axial compressive capacity of the composite column is shown in Figure 23. When no interlocking key is provided, load transfer between the aluminum alloy frame and the foam concrete relies primarily on interfacial adhesion and friction. Due to the low interfacial shear stiffness, relative slip readily occurs under axial compression, resulting in the aluminum alloy frame carrying most of the axial load independently and preventing effective composite action. Moreover, the foam concrete offers limited restraint against the local buckling of the aluminum alloy frame. Consequently, the axial compressive capacity is the lowest in this configuration.

When one or two interlocking keys are introduced, the load-transfer mechanism is substantially improved. The mechanical interlock formed between the aluminum alloy frame and the foam concrete enhances the interfacial shear resistance and facilitates more efficient axial load sharing. The presence of the interlocking keys increases the overall stiffness and stability of the composite column, allowing for better utilization of the material strengths. However, further increasing the number of interlocking keys introduces additional openings and geometric discontinuities within the concrete, which leads to a reduction in load-carrying capacity. Therefore, the optimal axial performance is achieved when the composite column is configured with two interlocking keys.

### 5.3. Aluminum Alloy Frame Thickness

**Figure 24:**
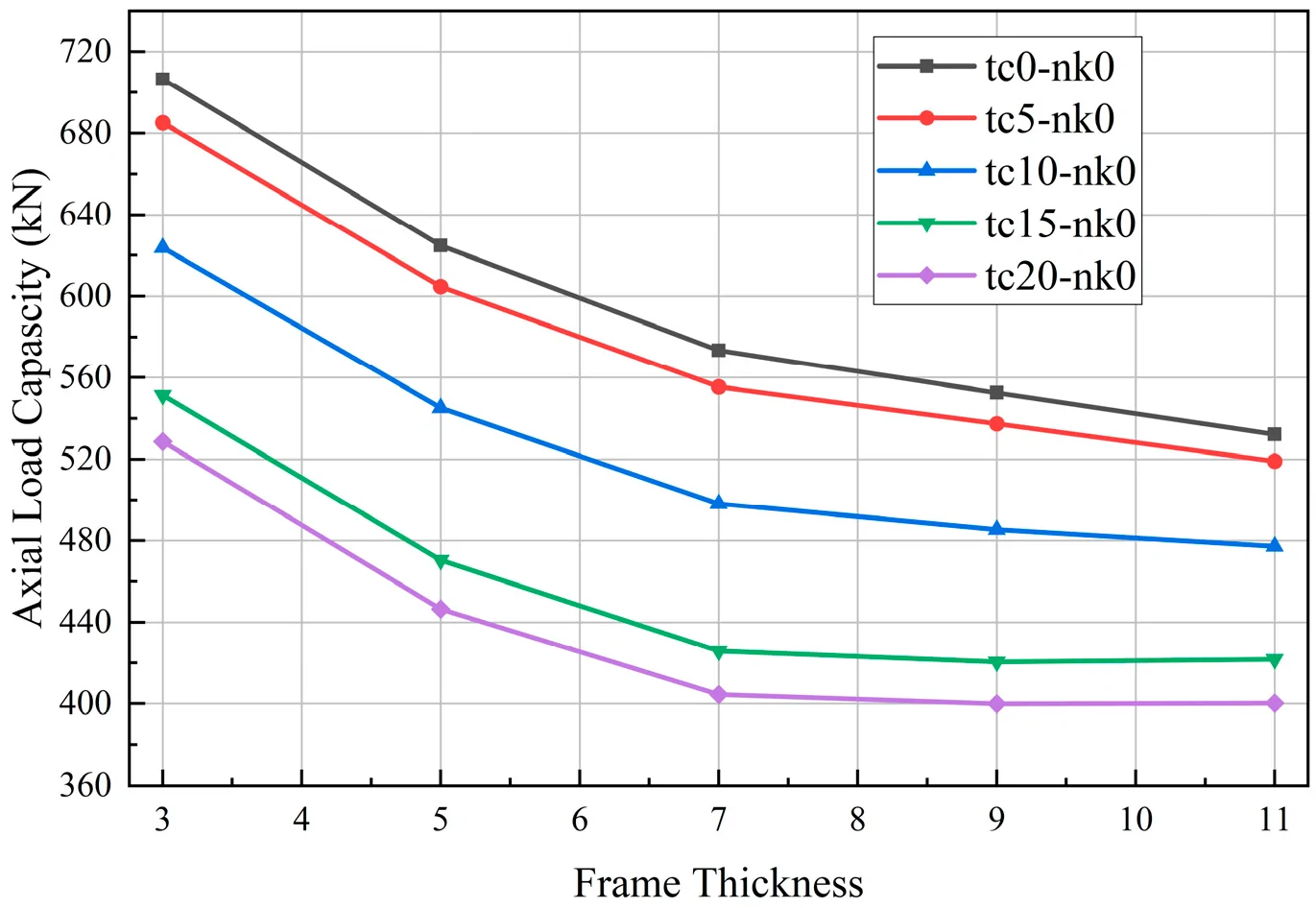
Influence of the Frame thickness. Note: *n_k_* denotes the number of interlocking keys, *n_k_* represents the concrete cover thickness, and *t_a_* indicates the frame thickness.

The influence of the aluminum alloy frame thickness on the axial compressive capacity of the composite column is shown in Figure 24. When the frame thickness is relatively small, an effective cooperative load-bearing mechanism is developed between the aluminum alloy frame and the foam concrete, allowing the load-carrying potential and confinement effect of the foam concrete to be fully mobilized, thereby yielding a higher axial capacity. As the frame thickness increases, the axial load is gradually dominated by the aluminum alloy frame, while the contribution and confinement effect of the foam concrete diminish, leading to a reduced composite action and a corresponding decrease in axial compressive capacity. This trend indicates that an optimal range of frame thickness exists, and an excessively large thickness is unfavorable for achieving the overall load-bearing advantages of the composite column.

## 6. Conclusions

This study examined the hypothesis that the embedded aluminum alloy frame and foam concrete can develop composite action under axial compression, and that this interaction is influenced by the interlocking keys, concrete cover thickness, and aluminum alloy frame thickness. The main findings are given as.

(1) Under axial compression, cracks first appear along the diamond-shaped diagonal braces of the middle column, while the middle column surfaces without diagonal braces exhibit only minor, fine cracks. As the axial load increases, the cracks along the diagonal-braced surfaces of the middle column gradually lengthen and widen. When the load reaches 600 kN, cracks also develop along the diagonal-braced surfaces of the top and bottom columns. Under axial compression, the middle column is more prone to cracking, with cracks tending to propagate along the diagonal-braced surfaces.
(2) In the initial loading stage, the lateral strain of the outer concrete generally exhibits negative values, indicating a compressive state. However, as the load increases, the diamond-shaped diagonal braces in the aluminum alloy frame, being geometrically flexible, cause slight lateral deformation of the vertical aluminum members, which significantly reduces their semi-passive confinement effect on the surrounding concrete. The longitudinal strain of the concrete at the 1/6, 1/2, and 5/6 heights of the composite column can be divided into two stages: the densification stage and the elastic stage. The strain evolution closely follows the trend of the axial load–displacement curve, indicating that, in the early loading phase, the foam concrete, as the primary compressive component, gradually increases in stiffness within a certain load range.
(3) Based on the finite element parametric study, within the investigated parameter range, the composite column with two interlocking keys exhibited the highest simulated axial load capacity. This result indicates that mechanical interlocking can improve composite action between the aluminum alloy frame and foam concrete. However, this optimal configuration was obtained from numerical analysis and should be further verified by additional full-scale tests.

## Figures and Tables

**Table 1:** Nominal chemical composition of 6005A-T6 aluminum alloy.

Element	Si	Mg	Fe	Mn	Cr	Cu	Zn	Ti	Al
Content/%	0.50–0.90	0.40–0.70	≤0.35	≤0.50	≤0.30	≤0.30	≤0.20	≤0.10	Balance

**Table 2:** Quasi-static tensile test results.

	Yield Strength (Extensometer)/MPa	Yield Strength (Strain Gauge)/MPa	Ultimate Tensile Strength/MPa
1	209.8	229.9	254.9
2	209.9	230.0	255.1
3	210.0	230.2	254.9
4	215.0	230.2	270.1
5	220.1	229.9	265.0
6	225.2	230.0	269.9

**Table 3:** Material properties of the aluminum alloy.

Nominal Yield Strain	Ultimate Tensile Strain	Elastic Modulus	Hardening Coefficient	Elongation
$\sigma_{0.2}=221\mathrm{MPa}$	$\sigma_{u}=262\mathrm{MPa}$	*E* = 70 GPa	*n* = 18	$\varepsilon_{f}=14.5$

**Table 4:** Approximate comparison of axial bearing capacity.

Method	Bearing Capacity/kN	Difference from Test/%
Experimental result	664.6	\
Finite element result	623.8	6.14
Simplified equivalent-area method	614.3	7.57

**Table 5:** Comparison of experimental and FE results.

	Test Peak Load/kN	Fe Peak Load/kN	Peak Load Deviation/%	Mean Relative Error/%
Figure 21a	623.8	659.5	5.73	6.62
Figure 21b	623.8	664.6	6.55	5.56
Average value	623.8	662.1	6.14	6.09

## Data Availability

Dataset available on request from the authors.
